# User experiences and perceptions on the use of digital health technologies in the management of type 2 diabetes: an integrative systematic review

**DOI:** 10.3389/fcdhc.2026.1750055

**Published:** 2026-02-03

**Authors:** Roberto Saraguro Betancourt, Mabely T. Mina Caicedo, Judith Francisco-Pérez

**Affiliations:** 1 Pontificia Universidad Catolica del Ecuador - Sede Esmeraldas, Esmeraldas, Ecuador; 2 Pontificia Universidad Católica del Ecuador, Quito, Ecuador

**Keywords:** barriers and facilitators, mobile applications, self-management, type 2 diabetes, user experiences

## Abstract

**Introduction:**

Type 2 diabetes mellitus (T2DM) represents a global public health problem. In Ecuador, it is the second leading cause of death among women and the third among men. Digital health technologies, including mobile applications, messaging platforms, and web-based tools, have emerged as promising interventions for managing this condition, although a gap remains between their theoretical potential and their effective implementation in clinical practice.

**Objective:**

To explore and synthesize the available evidence on the experiences, perceptions, barriers, and facilitators reported by adult users with type 2 diabetes mellitus regarding the use of digital health technologies for managing their disease in community or outpatient settings.

**Methodology:**

An integrative systematic review of literature published between 2015 and 2025 was conducted in PubMed, Scopus, and Google Scholar. Qualitative, quantitative, and mixed-methods studies in English and Spanish involving adults using mobile applications for T2DM were included. Methodological quality was assessed using the Johns Hopkins Evidence-Based Practice model.

**Results:**

A total of 66 studies were analyzed, with qualitative designs predominating (n=28), followed by randomized clinical trials (n=13) and experimental studies (n=14). Most studies were rated as high (n=35) or good quality (n=28). Experiences were grouped into three categories: positive (usefulness, satisfaction, empowerment, educational support), conditioning factors (individual, contextual, and design-related), and barriers (technological, usability, personal). Perceptions focused on usefulness, usability, and impact on self-care. Facilitators included simple design, personalization, professional support, cultural adequacy, and motivation.

**Conclusions:**

Digital health technologies promote self-management and glycemic control, although barriers persist that limit their sustained adoption.

**Systematic Review Registration:**

https://www.crd.york.ac.uk/prospero/, identifier CRD420251241989.

## Introduction

Type 2 diabetes mellitus (T2DM) poses a significant challenge to healthcare systems, affecting more than 537 million people worldwide, with projections indicating an increase of up to 51% by 2045 (1). Its prevalence ranges from 2% to 20% depending on the region, with the highest rates observed in Southeast Asia and the Eastern Mediterranean, and a notable increase in the Americas in recent decades (2–5).

In Ecuador, T2DM was the second leading cause of death among women and the third among men between 2016 and 2017 (4, 6). By 2018, between 7.1% and 7.8% of the Ecuadorian population had the disease, with a prevalence that increases significantly with age: from 2.7% among people aged 10 to 59 to 15.2% in the 60–64 age group (6). The coastal provinces and the island region have the highest rates, with a disproportionate impact on women (6, 7).

T2DM requires innovative self-management strategies, and digital health technologies—including mobile applications, messaging platforms, and web-based interventions—have emerged as promising tools for managing this condition (8), offering functionalities such as glucose monitoring, nutritional tracking, medication reminders, and diabetes education (9–11). However, a significant gap remains between the theoretical potential of these technologies and their effective adoption in clinical practice (12).

While some users report improvements in self-control and management of their condition, others face technological barriers, usability issues, and low satisfaction with app interfaces (13, 14). Evidence suggests that factors such as age, educational level, professional recommendation, and specific app features influence the user experience (15).

Despite the growing development of digital health technologies designed to support T2DM management, there is still limited understanding of user experiences from the perspective of patients themselves. Unmet specific needs, multidimensional barriers to implementation, contextual facilitators, and factors that contribute to sustained adherence are addressed only fragmentarily across individual studies (12, 15).

However, no evidence has been found that appropriately systematizes these findings, which prevents the development of a comprehensive understanding of the effectiveness and acceptability of these digital interventions (16). This absence of synthesis represents the central problem addressed by the present systematic review, which aims to consolidate available knowledge and provide guidance for future digital health strategies targeted at individuals with T2DM.

While previous systematic reviews have primarily focused on the clinical effectiveness of digital interventions for T2DM or specific technological platforms, there is a notable gap in integrative syntheses that comprehensively examine user-reported experiences, perceptions, barriers, and facilitators across diverse digital health technologies. The relevance of this review lies in the growing and critical need to optimize digital health interventions for patients with T2DM (17), considering the current global epidemiological landscape and the rapid transformations occurring in health service delivery (3). Contemporary scientific evidence shows that digital health technologies may not be clear or suitable for all users, which poses communication risks and may lead to complications associated with poor glycemic control (18).

From an epidemiological perspective, T2DM affects more than 537 million people worldwide according to data from the International Diabetes Federation (IDF) (1), with alarming projections indicating an increase of up to 51% by 2045, representing an estimated global healthcare expenditure of approximately 966 billion USD annually (3). This demographic and economic reality calls for stable, cost-effective, and sustainable long-term strategies (19). Di gital health technologies represent a particularly promising potential solution, especially considering that 85% of adults globally own mobile devices with advanced connectivity features (15). The technological context in Ecuador is also promising, with 83.7% internet coverage, 13.5 million social media users, and a digital health market projected to grow by 7.08% between 2025 and 2029 (20).

On the other hand, recent scientific literature shows contradictory results. While qualitative studies indicate that experienced users perceive improvements in self-control and adherence (13, 14), usability research reveals greater difficulties among older adults—highlighting the importance of understanding user experiences (11, 21).

Synthesizing the existing knowledge will allow the identification of critical elements for designing more effective applications and informing digital health policies. For Ecuador, this is particularly relevant given the unique challenges of local diabetes control and the need for culturally appropriate interventions that consider urban–rural disparities (20, 22). Likewise, it will help maximize the impact of interventions in the post-pandemic context, where digital tools have gained increasing relevance within health systems (23, 24).

Research question: What experiences, perceptions, barriers, and facilitators are reported in scientific literature regarding the use of digital health technologies by adults with type 2 diabetes for managing their disease in community or outpatient settings?

Objective: To explore and synthesize the available evidence on the experiences, perceptions, barriers, and facilitators reported by adult users with type 2 diabetes mellitus concerning the use of digital health technologies for managing their disease in community or outpatient settings.

## Methodology

This integrative systematic review was conducted and reported in accordance with the Preferred Reporting Items for Systematic Reviews and Meta-Analyses (PRISMA) 2020 guidelines. The review protocol was registered in PROSPERO (Registration number: CRD420251241989).

Methodological framework: This review followed the integrative review methodology proposed by Whittemore and Knafl (25), which allows for the systematic synthesis of diverse research designs (qualitative, quantitative, and mixed methods) to provide a comprehensive understanding of complex phenomena. This five-stage framework includes: (1) problem identification, (2) literature search, (3) data evaluation, (4) data analysis, and (5) presentation of findings. Each stage was rigorously implemented as described below to ensure transparency and reproducibility.

### Type of study (stage 1: problem identification)

Following Whittemore and Knafl’s framework, we first identified the problem and defined the purpose of this integrative review. The central problem was the limited understanding of user experiences with digital health technologies for T2DM management, despite their growing development and theoretical potential. An integrative literature review was conducted with the aim of compiling and analyzing empirical studies—qualitative, quantitative, or mixed methods—that explore the phenomenon of digital health technology use among adults with type 2 diabetes. The research question and objective were clearly formulated to guide the subsequent stages (see Introduction). Data synthesis was carried out from a qualitative perspective, allowing for an in-depth understanding of the experiences, perceptions, barriers, and facilitating elements related to the use of these digital tools in the daily management of the disease.

Study selection criteria: Studies published between 2015 and 2025, written in Spanish or English, available in full text, and involving adult individuals (≥18 years) diagnosed with type 2 diabetes mellitus were included. Eligible studies were those in which the central focus of analysis was the use of digital health technologies (as defined below) by users in the context of managing their chronic condition. This includes digital tools aimed at supporting monitoring, tracking, education, self-care, or treatment.

Definition of digital health technologies: For this review, digital health technologies encompass any technology-mediated intervention designed to support diabetes self-management through digital platforms. This includes: a) native smartphone applications, b) SMS/text-messaging programs, c) instant messaging platforms (e.g., WhatsApp chatbots), d) web-based platforms (including both mobile-accessible and desktop platforms), e) patient portals, and, f) social media-based interventions. This broad definition reflects the contemporary landscape of digital health interventions and acknowledges that patients often use multiple interconnected digital tools in their diabetes management, accessed through various devices including smartphones, tablets, and computers.

Likewise, documents that did not report empirical results were excluded, such as theoretical reviews, editorials, protocols, or conference abstracts, as well as studies focused on pediatric populations, individuals with type 1 diabetes or gestational diabetes, and studies involving professional groups without direct participation from patients.

### Stage 2: literature search

Search strategy: The search for studies was conducted in three electronic databases: PubMed (23 August 2025), Scopus (28 August 2025), and Google Scholar (31 August 2025).

PICo components guided the search strategy: P (Population): Adults with type 2 diabetes mellitus; I (Phenomenon of Interest): Use of digital health technologies (including mobile applications, messaging platforms, and web-based tools) for diabetes management; and Co (Context): Everyday settings such as home, community, or outpatient care.

Construction of the search strategy: Specific search equations were developed for each database, using both controlled vocabulary (such as MeSH or DeCS) and free-text descriptors. Boolean operators were used to establish relationships between terms and ensure efficient and comprehensive search. Complete search strategies for all databases, including specific terms, operators, filters, and number of results retrieved are provided in **Supplementary File 1**.

For Google Scholar, results were ordered by relevance, and the first page (12 results) was screened. Given the highly specific search terms and comprehensive coverage through PubMed and Scopus, Google Scholar served as a supplementary source to identify potential grey literature or regional publications not indexed in the primary databases.

Forward and backward citation tracking was performed for all included studies using Google Scholar and Scopus citation tools. Reference lists of included studies were also manually screened. These supplementary search methods did not yield additional articles meeting the inclusion criteria within the specified publication period.

Procedure for study selection: The results obtained were exported to the Mendeley reference management tool, where duplicates were removed. Title and abstract screening were conducted independently by two researchers. Articles that met the inclusion criteria progressed to full-text review. Any discrepancies were resolved through consensus or by consulting a third researcher.

Data recording and organization: An extraction matrix was developed in Excel to systematically collect the following information: a) General data: authorship, year, country, and study design; b) Description of the level and quality of the evidence; c) Results: experiences, perceptions, barriers, and facilitators related to the use of digital tools for diabetes management.

### Stage 3: data evaluation

Methodological appraisal of the studies: The methodological quality of included studies was assessed using the Johns Hopkins Evidence-Based Practice (JHNEBP) model appendices. This model classifies evidence according to two dimensions:

1. Level of evidence (based on study design): Level I = experimental studies and randomized controlled trials (RCTs); Level II = quasi-experimental studies; Level III = non-experimental, qualitative, and mixed-methods studies; Level IV = expert opinion; Level V = literature reviews.
2. Quality rating (based on methodological rigor and consistency of findings):
  • High quality (A): Consistent, generalizable results with sufficient sample size, adequate control of confounders, definitive conclusions, and consistent recommendations based on comprehensive literature review that includes thorough reference to scientific evidence.
  • Good quality (B): Reasonably consistent results with sufficient sample size, some control of confounders, definitive conclusions, and reasonably consistent recommendations based on comprehensive literature review that includes some reference to scientific evidence.
  • Low quality (C): Inconsistent results, insufficient sample size, little or no control of confounders, conclusions cannot be drawn, and recommendations based on limited review or inconclusive evidence.

Quality assessment was performed independently by two reviewers, with discrepancies resolved through consensus or consultation with a third reviewer. For each study, reviewers evaluated specific criteria including sample size adequacy, clarity of research question/objective, appropriateness of study design, data collection methods, analysis procedures, reporting of limitations, and consistency between results and conclusions.

The JHNEBP model was selected as it allows for the appraisal of diverse study designs (qualitative, quantitative, and mixed methods) within a single framework, which aligns with the integrative nature of this review. While design-specific tools (e.g., Cochrane Risk of Bias tool for RCTs, CASP for qualitative studies) provide more granular assessment, the JHNEBP model enabled standardized comparison across the heterogeneous body of evidence. This approach has been previously validated in integrative reviews synthesizing multiple methodologies. However, we acknowledge in the limitations section that the use of design-specific appraisal tools could have provided more detailed quality assessment for individual study types.

A complete summary table of evidence level and quality ratings for all 66 included studies is provided in **Supplementary File 2**.

### Stage 4: data analysis and interpretation

Data analysis and interpretation: Extracted data were processed using qualitative thematic analysis following an iterative and systematic approach. The analysis was conducted by two independent reviewers using Microsoft Excel for data organization and coding management.

The thematic analysis employed a predominantly inductive approach, allowing categories and subcategories to emerge directly from the data. While the broad domains of interest (experiences, perceptions, barriers, and facilitators) were predetermined by the research question, all subcategories and themes within these domains emerged through open coding without imposing a pre-existing theoretical framework. This inductive approach ensured that the synthesis remained grounded in the user-reported evidence from the included studies.

The coding process followed these steps:

1. Familiarization: Both reviewers independently read all included studies to gain overall understanding
2. Initial coding: Open coding was performed independently by both reviewers, systematically identifying all user-reported experiences, perceptions, barriers, and facilitators
3. Code comparison and consensus: Reviewers met regularly to compare codes and discuss interpretations. Discrepancies were resolved through discussion until consensus was reached. When agreement could not be reached, a third researcher was consulted to make the final decision
4. Category development: Related codes were grouped into categories and subcategories through constant comparison across studies
5. Theme refinement: Categories were iteratively reviewed and refined to ensure internal consistency and clear distinction between categories
6. Pattern identification: Common patterns, variations, and significant divergences across studies, contexts, and populations were identified and documented

Integration of quantitative and qualitative evidence: This integrative review synthesized findings from diverse study designs (qualitative, quantitative, and mixed methods). Quantitative outcomes from experimental studies and RCTs (e.g., satisfaction ratings, adherence percentages, hemoglobin A1c changes, usability scores) were narratively integrated into the thematic framework to enrich and contextualize the qualitative themes. For example, qualitative descriptions of “improved glycemic control” were complemented with quantitative data showing specific HbA1c (hemoglobin A1c) reductions from RCTs. Similarly, user-reported barriers were triangulated with quantitative measures of app discontinuation rates or low engagement metrics. Mixed-methods studies were not analyzed as a separate category; instead, their findings (both qualitative and quantitative) were integrated into the qualitative thematic framework. This convergent synthesis approach allowed us to capture both the experiential depth of user perspectives (from qualitative data) and the measurable outcomes (from quantitative data), providing a comprehensive understanding of digital health technology use in T2DM management.

### Stage 5: presentation of findings

The findings were structured into a narrative that reflects the diversity of user experiences, highlighting their experiences, perceptions, barriers, and facilitators in the use of mobile apps for the management of T2DM.

## Results

**Figure 1:**
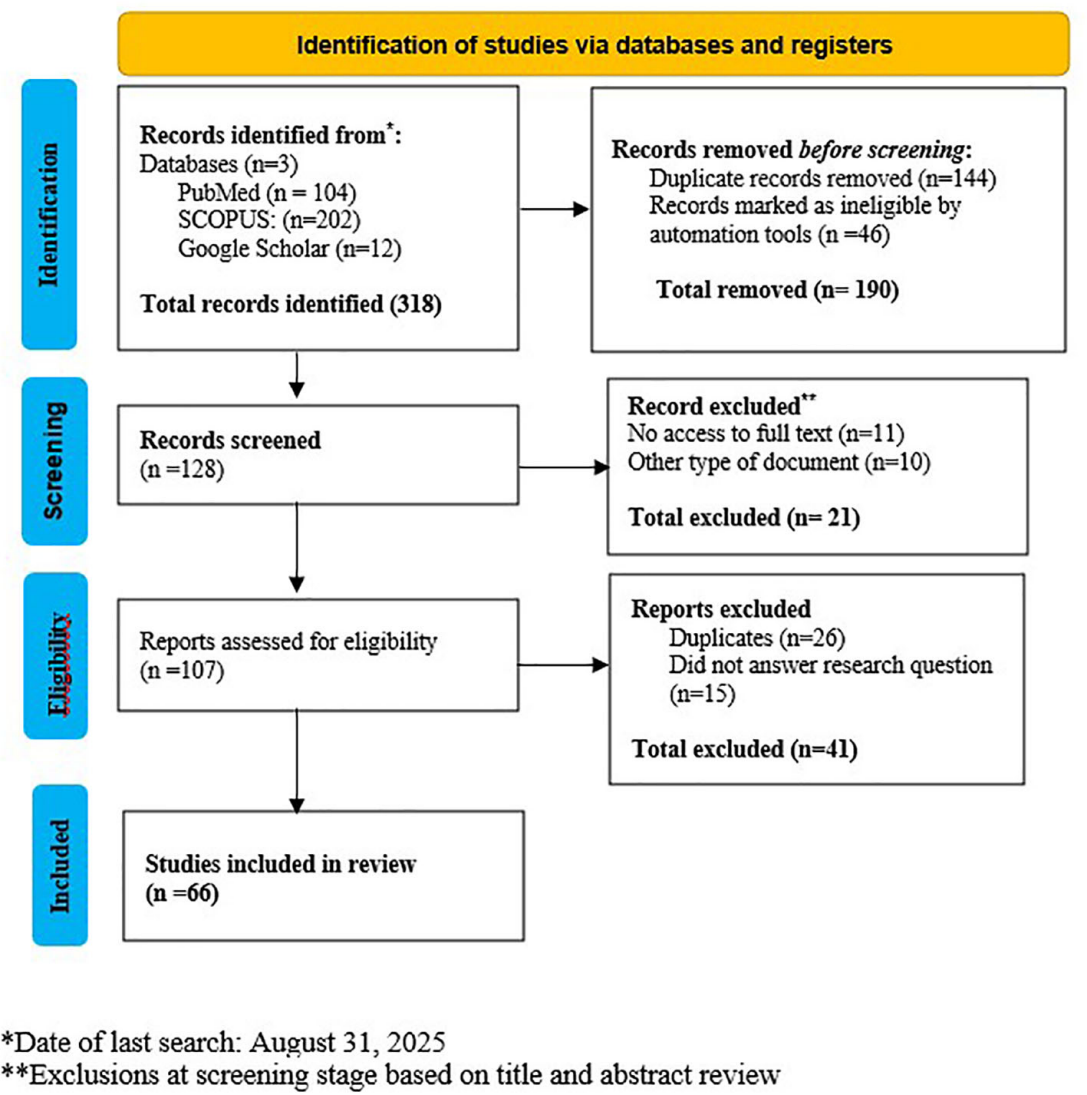
PRISMA flow diagram.

A total of 66 studies were included in this integrative systematic review (**Figure 1**). Most articles came from the Scopus database (n = 45), followed by PubMed (n = 21). Regarding methodological design, qualitative studies predominated (n = 28), along with randomized clinical trials (RCTs, n = 13) and quantitative experimental designs (n = 14), while mixed methods (n = 6) and observational studies (n = 5) were less represented. In terms of evidence quality and level, most studies were classified as high quality (n = 35) or good quality (n = 28), with a predominance of level III studies (n = 32), followed by level II (n = 18) and level I (n = 16).

With respect to geographic distribution, the studies were conducted mainly in the United States (n = 15), China (n = 7), and the United Kingdom (n = 5), while other publications originated from European, Asian, and Latin American countries to a lesser extent. Finally, an upward trend in scientific production has been observed since 2019, with a peak in 2022 (n = 13) and continued output in 2023 (n = 7) and 2024 (n = 11).

### Quantitative outcomes from experimental studies and RCTs

A total of 27 studies employed experimental designs (n=14) or randomized controlled trials (n=13) and reported quantitative outcomes related to the effectiveness of digital health interventions. The primary outcomes assessed across these studies included glycemic control (HbA1c), self-management behaviors, medication adherence, quality of life, and user engagement metrics.

Glycemic control (HbA1c): Among the 27 experimental/RCT studies, 19 measured HbA1c as a primary or secondary outcome. Of these, 11 studies (57.9%) reported statistically significant reductions in HbA1c favoring the digital health intervention. The magnitude of HbA1c reduction ranged from -0.30% to -1.3 percentage points, with reductions typically between -0.5% and -0.9%. Notable examples include Koot et al. (73) reporting a -1.3-percentage point reduction (p<.001), Caballero et al. (80) showing a significant difference between intervention (3.7% reduction) and control (2.6% reduction) groups (p=.006), and Sze et al. (52) demonstrating a -0.79% reduction (p=.0001). Studies with longer follow-up periods (≥6 months) generally showed sustained improvements in glycemic control [Caballero et al. (80), Feng et al. (26), Hu et al. (53)].

Self-management behaviors and adherence: Eighteen studies assessed self-management behaviors including medication adherence, dietary habits, physical activity engagement, or glucose self-monitoring using validated scales or objective measures. Of these, 13 studies (72.2%) reported significant improvements in at least one self-management domain. Medication adherence showed improvements in multiple studies [Caballero et al. (80), Newhouse et al. (34), Yang et al. (64)], while physical activity and dietary behaviors demonstrated significant positive changes in studies employing behavioral interventions [Feng et al. (26), Horner et al. (32), Leon et al. (55), Sze et al. (52)]. Feng et al. (26) reported significant improvements across multiple self-care activities including diet (β=0.34), exercise (β=0.46), and glucose self-monitoring (β=0.42). Self-monitoring compliance also improved notably, with Tang et al. (50) reporting 91.0% vs 59.7% SMBG compliance at 3 months in intervention versus control groups.

Quality of life and patient-reported outcomes: Eight studies evaluated quality of life, diabetes distress, or self-efficacy using standardized instruments. Significant improvements were reported in 5 studies (62.5%), particularly in domains related to diabetes-specific distress and self-efficacy for diabetes management [Caballero et al. (80), Krall et al. (14), Leong et al. (51), Poduval et al. (81)]. Caballero et al. (80) demonstrated improvements in diabetes-related quality of life (EsDQOL) and patient experience of care (IEXPAC), while Krall et al. (14) showed reductions in diabetes distress scores among intervention participants.

User engagement and satisfaction: Intervention studies consistently reported high initial user satisfaction rates, with multiple studies showing satisfaction scores above 80% [Hu et al. (53), Hu et al. (84), Krall et al. (14), Leong et al. (51)]. For example, Krall et al. (14) reported that 85.1% of users would recommend the app, 81.5% found it useful, and 85.2% found the interface attractive. Hu et al. (53) demonstrated a retention rate of 97% and mean satisfaction of 9.9/10. However, engagement metrics varied considerably across studies, with app usage typically declining over time. Blythin et al. (31) reported retention rates of 27.8% at 1 month and 10.3% at 6 months, while Batch et al. (28) found that 50.2% of participants did not download the app, and among those who did, only 48.0% completed all levels. This pattern of initial high engagement followed by declining use was observed across multiple studies, particularly after the first 4–8 weeks of intervention [Blythin et al. (31), Bults et al. (48), Kuo et al. (37), Maharaj et al. (45), Mash et al. (33)].

A detailed summary table of all experimental studies and RCTs, including study design, sample size, intervention type, duration, and primary quantitative outcomes with effect sizes, is provided in **Supplementary File 3**.

### Results of users’ experiences

In the analysis of user experiences, three main categories emerged: positive experiences, conditioning factors, and barriers. For each of these, subcategories were organized to group the findings from the evidence.

As shown in **Table 1**, several studies indicated that the applications were perceived as useful, motivating, and relevant to daily life, facilitating self-management and treatment adherence (14, 23, 29, 31, 36, 42). Users valued aspects such as personalized content, educational and motivational support, and digital accompaniment—elements that increased satisfaction and commitment to their self-care (35, 44, 50).

**Table 1:** Reported experiences in the use of digital health technologies for type 2 diabetes.

Category	Subcategory	Evidence
Positive experiences	Perceived usefulness	Improvement in self-management, reduction in HbA1c, and increased adherence (26, 27).
	Satisfaction and acceptance	High satisfaction, relevance to daily life, and recommendation to others (14, 28, 29).
	Empowerment	Visualization of health data and feedback that facilitates behavioral change (30, 31).
	Educational and motivational support	Interventions using messages, chatbots, and multimedia that enhanced motivation and self-care (32–35).
Conditioning factors	Individual factors	Self-care style (self-directed vs. passive) (36, 37). Motivation and health literacy (38, 39).
	Contextual factors	Family support and professional recommendation as key elements for continued use (40–42).
	Design-related factors	Intuitive interfaces, personalization, and variety of functions (43, 44).
Barriers	Technological barriers	App complexity, usability failures (45–47).
	User-related barriers	Information overload, data entry fatigue, and sustained demotivation (41, 48, 49).
	Contextual barriers	Access limitations, poor integration with other technologies, difficulties among older adults (39).

Various studies demonstrated that these tools promoted positive changes in diet, physical activity, and glycemic control, with significant reductions in HbA1c and greater confidence in managing the disease (26, 27, 30, 51, 52). The use of multimedia resources such as videos and text messages was also identified as an effective motivational reinforcement (32–34, 53).

The experiences showed differences according to the self-care style: more self-directed users were more proactive and consistent, whereas others demonstrated lower levels of engagement. In this regard, personal motivation, social support, and recommendations from healthcare professionals were key determinants for sustained use (36–38, 41, 54, 55).

Finally, relevant barriers were identified, including information overload, interface complexity, message repetitiveness, data entry fatigue, and limitations in digital literacy, particularly among older adults (39, 45–49).

In this part of the analysis, three main categories emerged: perceived usefulness, perceived usability, and perceived impact on self-care, organized into eight subcategories. Perceived usefulness was expressed through positive evaluations, ambivalent perceptions, and feelings of confidence and safety. Usability included perceived ease of use and perceived limitations. Finally, the impact on self-care encompassed motivation/adherence and variability in engagement. These findings are summarized in **Table 2**.

**Table 2:** Perceptions reported in the use of digital health technologies for type 2 diabetes.

Category	Subcategory	Evidence
Perception of usefulness	Positive appraisal	Apps are perceived as useful for improving self-management, adherence, and glycemic control (14, 27, 30, 35, 38, 41, 43, 44, 50, 52, 53, 56–68)
	Ambivalent or critical	Doubts about real benefits, preference for human contact, sense of loss of autonomy (47–49, 54, 69, 70)
	Confidence and security	Increased confidence in disease management; feelings of safety and support (33, 42, 71, 72)
Perception of usability	Ease of use	Apps perceived as easy, attractive, and accessible (14, 36, 73)
	Usability limitations	Reports of slowness, repetitiveness, and difficulties among older adults (32, 39, 45)
Perception of impact on self-care	Motivation and adherence	Apps motivated changes in diet, physical activity, and medication adherence (46, 51, 55, 74, 75)
	Variability in engagement	Initial positive intention that decreases over time; depends on personal motivation and social support (34, 37)

The perception of usefulness stood out as the most frequent, highlighting that the applications were viewed as valuable for improving self-management, glycemic control, and treatment adherence (14, 27, 30, 35, 38, 41, 43, 44, 50, 52, 53, 56–68). However, some participants expressed ambivalent perceptions, showing doubts about the real benefits of the applications or their compatibility with the need for human contact (47–49, 54, 69, 70).

Regarding perceived usability, most participants agreed that the applications were easy to use, attractive, and accessible (14, 36, 73). Nevertheless, design limitations, slowness, lack of interactivity, and difficulties among older adults were reported, which reduced satisfaction and continuity of use (32, 39, 45).

Finally, in terms of perceived impact on self-care, users highlighted that the apps fostered motivation, treatment engagement, and the adoption of healthier habits (46, 51, 55, 74, 75). Variability in engagement over time was reported, with some users describing initial positive intention followed by declining use (34, 37).

### Barriers to the use of mobile applications for type 2 diabetes

As shown in **Table 3**, four categories of barriers were identified in the analysis of the studies: technological, design and usability, individual, and contextual/social, organized into various subcategories. Technological barriers included lack of internet access or adequate devices, as well as technical failures and connectivity problems (31, 33, 58, 76). Design and usability barriers were related to complex interfaces, information overload, slowness, repetitive messages, and limited personalization, which generated frustration and abandonment (43, 45, 46, 48, 49).

**Table 3:** Barriers identified in the use of digital health technologies for type 2 diabetes.

Category	Subcategory	Evidence
Technological	Access and connectivity	Lack of internet, smartphones, or link reception; technical and connectivity failures (31, 33, 58, 76)
	Technical failures	System overload, automatic errors, compatibility issues, and battery problems (33, 74)
Design and usability	Complexity and overload	Interfaces that are not intuitive, confusing icons, excessive functions or repetitive messages (43, 45, 46, 48, 49)
	Limited personalization	Lack of adaptation to individual needs, decreasing motivation, and apps perceived as not very relevant (36, 41)
Individual factors	Literacy and skills	Limitations in digital/health literacy, lack of technological knowledge, cognitive or physical difficulties (27, 39, 56)
	Motivation and adherence	Decreased engagement over time, technology fatigue, forgetfulness, or sustained lack of interest (37, 59, 66)
Contextual and social	Stigma and social acceptance	Fear of being judged, cultural resistance, lack of trust in technology (49, 54, 77)
	Cultural and linguistic factors	Language barriers, non-adapted dietary plans, and cultural differences (40, 75)
	Socioeconomic factors	Costs, inequities in access, and lack of time due to work and family responsibilities (67, 78)
	Insufficient support	Absence of family, community, or professional support to sustain usage (36, 62)

Regarding individual barriers, the studies mentioned low digital and health literacy, cognitive or physical limitations, sustained lack of motivation, and difficulties integrating the app into daily routines (27, 39, 41, 56, 66). Finally, among the contextual and social barriers, stigma, lack of family or professional support, cultural and linguistic limitations, and socioeconomic factors that restrict adoption and sustained use of these technologies stood out. These difficulties were reported across multiple studies (35, 40, 67, 75, 77, 78).

### Facilitators of the use of mobile applications for type 2 diabetes

**Table 4** presents the results of the facilitators identified in the literature. These were organized into three main categories: 1) design and functionality of the intervention, 2) human and ecosystem support, and, 3) user-related factors and personal context. These categories were subdivided into eleven subcategories, which allow for a more precise description of the elements that favored the acceptance, adherence, and effectiveness of mobile applications in the management of type 2 diabetes.

**Table 4:** Identified facilitators of the use of digital health technologies for type 2 diabetes.

Category	Subcategory	Evidence
Design and functionality of the intervention	Simple usability and integration of functions	Access to logs, reminders, and data visualization facilitated self-management and engagement (14, 30, 31, 38, 41, 56, 58, 59, 77)
	Personalization and feedback	Personalized interactions, adapted content, and conversational agents reinforced adherence and motivation (14, 28, 36, 39, 44–46, 66)
	Interactivity and gamification	Videos, SMS, and gamification features served as motivators and reinforced healthy habits (32, 34, 55, 61, 75)
	Clinical integration and devices	Insulin titration, automatic synchronization, and comfortable devices improved confidence and adherence (27, 29, 52, 64, 79).
	Security and privacy	Perceived security and confidentiality favored trust in the use of the app (68, 70)
Human and Ecosystem Supports	Coaching and professional accompaniment	Guidance from professionals and bilingual health coaches strengthened trust, motivation, and adherence (27, 38, 52, 62, 63, 72)
	Professional recommendation and institutional collaboration	Professional recommendations and institutional support facilitated acceptance and continued use (29, 33, 80–83)
	Technical support and training	Digital training, technical support, and telephone assistance enabled adoption, even among users with low digital literacy (36, 62, 64, 65)
	Family channels and reach	WhatsApp, videoconferencing, and phone calls increased accessibility and facilitated follow-up, including in community settings (33, 48, 53, 55, 75, 76, 84, 85)
	Community networks and social support	The support of family members, communities, and social networks strengthened self-management, confidence, and resilience in coping with the disease (67, 69, 71, 74, 83, 86, 87)
User Factors and Personal Context	Motivation and Self-Efficacy	Personal motivation, perceived responsibility, and self-efficacy facilitated adherence and commitment (34, 37, 49, 54, 60, 63, 64, 68)
	Convenience and Habits	Flexibility, device portability, and integration with daily routines reinforced self-management (42, 54, 58, 76, 77)
	Technological Familiarity	Previous experience with mobile phones and internet use facilitated app acceptance (48, 50, 53, 57, 84, 85)
	Cultural and Linguistic Appropriateness	Culturally adapted messages, in the local language and using relatable examples, promoted acceptance (33, 40, 75, 80, 85)
	Family and Social Support	Support from family members, caregivers, and friends increased adherence, motivation, and sustained use (63, 67, 69)
	Agency and Confidence	Perceived control, anonymity, and a sense of safety strengthened confidence in using the applications and maintaining their use (68, 70, 74)

Design and functionality of the intervention: Several studies highlighted that simple usability and the integration of functions such as reminders, automated feedback, and data recording facilitated patient self-management and engagement (14, 30, 31, 38, 41, 56, 58, 59, 77). Personalization and feedback were perceived as key motivators, as they allowed content to be adapted to individual needs, supporting learning and self-confidence (14, 28, 36, 39, 44–46, 66). Likewise, interactivity and gamification increased motivation by incorporating playful elements and progress visualizations (32, 34, 55, 61, 75). Finally, integration with clinical care and other devices was identified as an important facilitator, as it improved adherence, optimized insulin titration, and strengthened the connection with healthcare teams (27, 29, 52, 64, 79).

Human support and ecosystem: Coaching and professional accompaniment, both through healthcare professionals and conversational agents or health coaches, was identified as a key facilitating factor by providing motivation, education, and cultural closeness (27, 38, 52, 62, 63, 72). Likewise, professional recommendation and institutional collaboration increased users’ trust and willingness to adopt these technologies (29, 33, 80–83). Technical support and initial training were also highlighted as factors that facilitated use, even among individuals with low digital literacy (36, 62, 64, 65). In addition, access to familiar and widely used channels such as WhatsApp, telephone calls, and videoconferences contributed to acceptance and adherence (33, 48, 53, 55, 75, 76, 84, 85). Finally, the role of community networks and social support was reported as an essential element for sustaining self-management, strengthening confidence, and reducing the sense of isolation (67, 69, 71, 74, 83, 86, 87).

User Factors and Personal Context: The facilitators were related to users’ disposition, motivation, and individual characteristics. Personal motivation and self-efficacy were identified as key determinants for maintaining engagement with the applications (34, 37, 49, 54, 60, 63, 64, 68). Convenience and daily habits, such as frequent mobile phone use and the ability to integrate the app into daily routines, were also noted as important facilitators (42, 54, 58, 76, 77). Prior technological familiarity with devices and applications increased acceptance and sustained use of these tools (48, 50, 53, 57, 84, 85). Likewise, cultural and linguistic appropriateness of the content supported user participation and adherence across different contexts (33, 40, 75, 80, 85). Close family and social support was another key facilitator that strengthened adherence and commitment to self-care (63, 67, 69). Finally, agency and confidence in using the technology enabled users to feel safer and more empowered in managing their condition (68, 70, 74).

## Discussion

This integrative systematic review included 66 studies encompassing a diversity of contexts, populations, and methodological approaches related to the use of digital health technologies for the management of T2DM. The characterization of these results shows that most investigations were conducted in community or outpatient settings, with interventions based on mobile applications, instant messaging, or social platforms. Although there is heterogeneity in the design of the interventions and in participant profiles, a common pattern emerges: the predominance of positive perceptions regarding the usefulness and motivation generated by the apps, alongside the identification of persistent barriers and facilitators that shape their acceptance and long-term sustainability.

The fact that most studies originate from developed countries highlights the need to generate evidence, where digital divides, health literacy levels, and structural conditions may influence the effectiveness and acceptability of these technologies. This lack of representation may limit the applicability of the findings to contexts with greater inequality in access to technology.

The reported barriers had important implications for long-term engagement and sustained use. Information overload, interface complexity, and data entry fatigue not only reduced immediate satisfaction but also compromised users’ commitment over time, particularly when compounded by low digital literacy in older adults. The variability in engagement observed across studies suggests that initial positive intention tends to decline when users lack sufficient personal motivation or social support. These patterns indicate that technological barriers interact with individual and contextual factors to shape long-term adherence—a finding that has important implications for intervention design and implementation strategies.

### Positioning findings within existing literature

Our findings both converge with and extend previous systematic reviews on digital health interventions for T2DM. Consistent with earlier reviews [Herrera et al. (12), Santana-Mejía & Romo (9)], we identified that digital technologies could improve glycemic control and self-management behaviors. Like the patterns documented by Blasco-Blasco et al. (2) in Latin America and the Caribbean, our review confirms that barriers such as low digital literacy, technological access limitations, and usability challenges remain persistent obstacles across diverse contexts. The declining engagement over time observed in our synthesis aligns with findings from Narindrarangkura et al. (21), who highlighted the challenge of sustained app use in diabetes management.

However, our review makes several novel contributions that distinguish it from previous syntheses. First, while prior reviews have focused predominantly on clinical effectiveness outcomes or specific technological platforms (e.g., continuous glucose monitoring, SMS interventions), our integrative approach encompasses the full spectrum of user-reported experiences across diverse digital health technologies, including mobile apps, messaging platforms, web-based tools, and social media interventions. This breadth allows for a more comprehensive understanding of the patient perspective across the digital health ecosystem.

Second, our review captures more recent evidence (2015-2025), including post-pandemic developments when digital health adoption accelerated globally. This temporal scope enables us to document emerging patterns such as the integration of conversational agents, gamification features, and hybrid human-digital support models that were less prominent in earlier reviews.

Third, our synthesis provides a more granular and structured categorization of barriers and facilitators organized across technological, individual, and contextual dimensions. This multi-dimensional framework goes beyond the predominantly technology-focused barriers identified in earlier reviews, revealing how personal motivation, social support, cultural appropriateness, and professional recommendation interact with technological features to shape user experience and sustained engagement.

Fourth, while most previous systematic reviews have drawn primarily from high-income country settings, our review intentionally sought evidence from diverse geographical contexts, including Latin American and other low- and middle-income countries (LMICs). Although representation from these regions remains limited in the published literature—a gap we explicitly acknowledge—our synthesis highlights the critical importance of cultural adaptation, linguistic appropriateness, and consideration of socioeconomic barriers such as connectivity issues and device access. These contextual factors, which may be less salient in high-income settings, emerge as fundamental determinants of effectiveness and acceptability in resource-constrained environments.

Finally, by synthesizing both the clinical effectiveness data from experimental studies and the rich qualitative experiences reported by users, our review bridges a gap between efficacy-focused and experience-focused literature. This convergent approach reveals a critical insight: while many interventions demonstrate clinical effectiveness (with 57.9% of RCTs showing significant HbA1c reductions), the user experience challenges—particularly declining engagement, usability frustrations, and preference for human contact—suggest that clinical efficacy alone is insufficient for real-world implementation success. This finding has important implications for intervention design, suggesting the need for hybrid models that combine technological efficiency with human support and personalization.

The increase in publications in recent years may be linked to the global momentum toward digital transformation in health, reinforcing the relevance of this study for guiding the design of contextually appropriate interventions.

The reported experiences suggest that digital health technologies may serve as useful tools for self-management, therapeutic adherence, and the improvement of lifestyle habits (26, 28, 30, 31, 36, 43). Participants particularly valued personalization, data visualization, and digital support—elements that enhanced satisfaction and engagement (36, 44, 50). However, differences associated with self-care style were also observed: those with greater self-direction were more consistent and proactive, while others discontinued use over time, underscoring the influence of motivation and social support (37, 38, 41).

Perceptions regarding the use of the apps consistently highlighted their ease of use, convenience, and contribution to glycemic control, although with nuances. While most participants considered these tools to facilitate self-care and increase awareness of their condition (14, 27, 29, 30, 55), critical perceptions also emerged, related to the lack of human contact, the effort required to record data, and doubts about the real impact on well-being (48, 49, 54, 66). These differences reflect that the perception of usefulness is influenced both by the user’s experience and by cultural, social, and personal factors.

The identified barriers were grouped into four main areas: technological, usability-related, individual, and contextual. Among the most frequent were information overload, non-intuitive interfaces, data entry fatigue, low digital literacy, and difficulties integrating the app into daily routines (30, 39, 41, 45, 48, 59). Contextual factors were also reported, such as socioeconomic inequities, connectivity problems, and cultural or linguistic barriers, which limited accessibility and adherence (40, 58, 76). These barriers suggest that the potential of digital interventions may be reduced if the users’ structural and social conditions are not adequately considered.

The facilitators complemented the perspective on obstacles, showing that simple usability, personalization, continuous feedback, and integration with the health system enhance the acceptance of digital health technologies (27–29, 36, 52). Likewise, intrinsic motivation, technological familiarity, social support, and recommendations from healthcare professionals emerged as key elements that promote continued use (33, 54, 55, 74). These results reinforce the notion that digital interventions are more successful when accessible technological resources are combined with human and community support.

Taken together, the findings suggest that digital health technologies have potential to improve self-management of type 2 diabetes; however, the evidence for their effectiveness remains limited by methodological constraints, short follow-up periods, and heterogeneity across studies. When effectiveness is observed, it appears to depend on the interaction between technological design, individual characteristics, and the sociocultural context. Positive experiences and perceived usefulness contrast with sustainability and accessibility barriers, which can be mitigated through facilitators related to human support and technological personalization.

### Strength and limitations of the evidence base

The findings must be interpreted with caution given several limitations of the underlying evidence. Many RCTs had small sample sizes (often <50 participants per arm) and short follow-up periods (mostly <6 months), limiting conclusions about long-term effectiveness. The heterogeneity in intervention design and outcome measurement across studies restricts the ability to identify which specific features are most effective. Additionally, many studies did not adequately report attrition rates, and the discordance between high satisfaction (>80%) and declining engagement (retention rates of 10-28% at 6 months in some studies) suggests satisfaction may not predict sustained use. Most evidence comes from high-income countries, with limited representation from Latin America, Africa, and South Asia, raising questions about generalizability to resource-constrained settings. These limitations underscore the need for cautious interpretation: while digital technologies show promise, the current evidence does not yet provide robust support for widespread implementation as stand-alone interventions.

## Conclusions

### Summary of core findings

The findings of this integrative systematic review show that digital health technologies have strong potential to strengthen self-management in T2DM, promoting therapeutic adherence, metabolic control, and the adoption of healthy behaviors. However, their effectiveness depends on the interaction between technological design, individual user characteristics, and the sociocultural context in which they are implemented.

### Limitations

Among the main limitations of the study are the inclusion of articles from only three databases, which may have restricted the breadth of the evidence; the language restriction to English and Spanish, which potentially excluded studies published in other languages; the heterogeneity of study designs and contexts, which limits direct comparability and precludes conducting a meta-analysis; and the conceptual heterogeneity of included digital health technologies (ranging from native mobile apps to SMS programs, web platforms, and social media interventions), which, while reflecting real-world practice, limits the ability to draw technology-specific conclusions about effectiveness; and the use of the JHNEBP model for quality appraisal, which, while appropriate for integrative reviews, may not have captured all potential sources of bias as comprehensively as design-specific tools (e.g., Cochrane Risk of Bias 2 for RCTs, CASP for qualitative studies).

### Implications for clinical practice and nursing

The findings suggest that nursing staff are strategically positioned to drive the effective adoption of digital health technologies among people with diabetes, as they typically lead ongoing education, support, and monitoring. In practice, this involves routinely incorporating a brief digital literacy assessment to tailor support, providing initial hands-on training and sustained technical support (with an emphasis on older adults or those with low digital literacy), and operating hybrid models that combine digital tools with regular human follow-up to maintain motivation, address difficulties, and monitor usage. It is also recommended to ensure that interventions are culturally and linguistically adapted to the individual’s context and to anticipate structural barriers through service-based solutions, such as device loan programs, connectivity availability in healthcare facilities, and simplified options for those with limited internet access or older equipment.

### Policy implications

The findings suggest that digital health strategies for diabetes should be designed with a focus on equity, integration, and sustainability. This involves creating financing mechanisms that recognize and reimburse the time health personnel invest in training, monitoring, and providing support related to digital tools; requiring interoperability and secure, bidirectional data exchange between applications and electronic health records to facilitate clinical decisions and avoid duplication; and investing in digital equity infrastructure to reduce connectivity gaps, subsidize devices, and strengthen community-based digital literacy programs, especially in rural or underserved areas.

Furthermore, the proposal suggests promoting co-creation with diverse end users through incentives or regulatory requirements that ensure apps address real needs and adopting “glocal” regulatory frameworks that establish national standards for quality, security, and interoperability, while allowing for local adaptations based on cultural context. Finally, it recommends supporting implementation science by funding studies and demonstration projects that evaluate integration strategies, implementation frameworks, and cost-effectiveness to guide scaling decisions.

### Research implications

Several critical research gaps emerged from this review. Future research should prioritize: 1) conducting more RCTs and implementation studies in Latin America, Africa, and other LMICs to understand how effectiveness and acceptability vary across diverse resource and cultural contexts; 2) designing studies with longer follow-up periods (≥12 months) to assess sustained engagement and long-term clinical outcomes, given that most current evidence is limited to 3–6 months; 3) incorporating theory-based frameworks (e.g., Technology Acceptance Model, Self-Determination Theory) to better understand mechanisms of adoption and sustained use; (4) examining which specific technological features and combinations are most effective, given current evidence heterogeneity; 5) investigating strategies to bridge the gap between high initial satisfaction and declining engagement; and 6) conducting mixed-methods studies that integrate quantitative effectiveness data with rich qualitative insights into user experience, particularly focusing on factors that support sustained use beyond the initial adoption phase.

## Data Availability

The original contributions presented in the study are included in the article/**Supplementary Material**. Further inquiries can be directed to the corresponding author.
