# Supplementary material for: User experiences and perceptions on the use of digital health technologies in the management of type 2 diabetes: an integrative systematic review
Source: Front Clin Diabetes Healthc. 2026 Feb 3;7:1750055. doi: 10.3389/fcdhc.2026.1750055 (PMC12909238; doi:10.3389/fcdhc.2026.1750055)
Supplement: Supplementary file 1 [file DataSheet1.pdf]

Article	Author (year)	Study design	Sample size	Type of intervention	Duration	HbA1c	Adherence/ behaviors	Quality of life (QoL)	Satisfaction/engagement
1	Pal et al. (2018)	Qualitative (4 focus groups)	20	NOT APPLICABLE (exploration of perceptions about web-based interventions for self-management)	Not applicable	Did not measure HbA1c; qualitative study on perception and experience of using digital interventions in T2D.	Addresses self-care behaviors based on perceived experience (without effect metrics).	No quantitative changes in QoL reported.	Qualitative engagement/satisfaction (barriers, facilitators, acceptability; no percentages).
2	Baptista et al. (2020)	Qualitative (substudy of a randomized clinical trial; semi-structured telephone interviews)	19	My Diabetes Coach (MDC) app with conversational agent "Laura" in personalized weekly sessions	Up to 12 months of prior use of the app (in the intervention arm)	Did not measure HbA1c; qualitative study ("perfect app") on needs/expectations in self-management apps.	Discusses self-care behaviors (reminders, education, follow-up) as desired needs, without quantitative results.	No quantitative improvements in QoL reported.	Usage/engagement preferences described qualitatively; no usage rates/percentages.

3	Baptista et al., 2020	Mixed methods (convergent design)	93 (intervention arm); 66 with 6-month follow-up; 19 interviews	MDC app with conversational agent (education and self-management support)	6 months (experience in a real-world environment)	They do report HbA1c as a clinical variable (at least baseline). Baseline: mean 7.3% (SD 1.5). They obtained it with consent from the general practitioner. This article (on the acceptability of the conversational agent) does not report reduction, significance, or ranges of change in HbA1c.	It does not report quantitative adherence results (or "significant improvements"); the focus is on acceptability/experience.	It does not report quality of life outcomes.	6-month survey: 86% describe the agent as helpful, 86% as friendly, 85% as competent, 73% as trustworthy, and 61% as likable. They also report associated emotions: motivated 44%, comfortable 36%, etc. In usage patterns, they report "total interactions with Laura" (mean 18; SD 15 in the intervention arm population; higher in subsamples) and that most interviewees were still using the app at the time of the interviews. No formal dropout/attrition rates are reported in this article.

4	Batch et al. (2021)	Pragmatic pilot study in a real-world setting (comparison: those who download vs. those who do not download)	201 (100 downloaded; 101 did not download)	Time2Focus app (structured program organized by levels)	12 weeks (12 levels) and measurement at 6 months	Yes. Change at 6 months: -0.41% (intervention) vs. -0.32% (control), P=.73 (not significant).	Yes. Not significant: non-adherence to medication -0.18 (P=.11) and physical activity 0.24 (P=.14).	QoL is not reported as a primary outcome in the reviewed abstracts.	High satisfaction: 4.56/5.00; NPS 62.5. Patterns: 50.2% did not download the app; among downloaders, 48.0% completed all levels; 18.6% reached level 6.
5	Blythin et al. (2023)	Retrospective (cohort; real-life data extracted from the platform)	2,783 registered; 1,245 activated users	myDiabetes app (digital self-management platform)	12 months (Nov 2019–Oct 2020)	It does not report HbA1c as a clinical outcome of the study; the focus is on large-scale usage/engagement analytics.	It does not report quantitative pre-post changes in adherence; it describes the use of educational/self-monitoring features (in terms of engagement).	No quantitative changes in QoL reported.	Engagement: activation 49.6%; retention 27.8% at 1 month and 10.3% at 6 months; high viewing of educational content (metrics in the study).

6	Boels et al. (2019)	Randomized clinical trial (block allocation; unblinded due to the nature of the intervention)	228 required (114 per group)	mHealth intervention (in-app messaging; education/support for self-management)	Main follow-up at 6 months (and 9 months for intervention only)	Yes. Adjusted intervention-control difference: -0.93 mmol/mol (equivalent to -0.08%), P=0.557 (not significant).	Yes. Self-care outcomes measured; no consistent effects (and reported under record/journal compliance).	Yes (multiple QoL/HRQoL scales): no relevant differences between groups.	Satisfaction/acceptability with percentages: motivation, recommendation, and usability reported in questionnaire; also SMS usage pattern (median 20 messages; range 1–123).
7	Bults et al. (2022)	Sequential explanatory design (mixed methods: web questionnaire + interviews)	103 questionnaires analyzed; 16 interviews	NOT APPLICABLE (explores barriers and facilitators to app use; does not implement intervention)	NOT APPLICABLE (perception/use study; not an intervention program)	Did not measure HbA1c; study focused on adoption/use and perceptions (not an effectiveness trial).	Addresses behaviors from perception/experience (without quantitative results).	No quantitative improvements in QoL reported.	Engagement/satisfaction described as drivers and barriers to use (qualitative; no percentages).
8	Burford et al. (2019)	Qualitative (semi-structured interviews)	28	Use of iPad and portal (Your Diabetes, Your Way) for decision support and self-management	9 months	Does not report HbA1c as a result; study focused on experience/use (qualitative).	Discute cambios/autogestión percibidos (medicación, dieta, control) sin cuantificación de efecto.	Discusses perceived changes/self-management (medication, diet, monitoring) without quantifying the effect.	Qualitative engagement: perceived usefulness, enablers/barriers; no percentages.

9	Caballero Mateos et al. (2025)	Multicenter, randomized, prospective, interventional	85 (control n=41; intervention n=44)	Education through social media and digital tools with "Digital Coach" (weekly and on-demand advice)	6 months of follow-up	Yes. HbA1c reduction: 3.7% intervention vs. 2.6% control; significant difference (P=.006).	Yes. Significant improvement in adherence/persistence: 13.8% intervention vs. -8.0% control.	Yes. Improvement in Diabetes Quality of Life Questionnaire (difference -13.7) and better care experience (IEXPAC).	Satisfaction/experience: improvement in perceived experience of care (IEXPAC) and satisfaction related to the program (detailed in results).
10	Cheng et al. (2022)	Qualitative exploratory study (semi-structured interviews)	20	NOT APPLICABLE (explore the use of official WeChat accounts for health information)	NOT APPLICABLE (interview duration: average 39.85 min; range 25–90)	Did not measure HbA1c; qualitative study (interviews) on the WeChat platform.	Behaviors addressed as expected needs/functions for self-care (diet, exercise, medication, monitoring).	No quantitative changes in QoL reported.	Qualitative engagement: "high willingness" to use; and 70% reported having used or currently using official accounts for diabetes management (usage profile).

11	Desveaux et al. (2018)	Qualitative assessment using a “realist evaluation” approach, semi-structured interviews	16 participants with T2DM (26 interviews)	Mobile app/web solution to support self-management in DM2	3 months	Yes. HbA1c was a quantitative outcome of the trial from which the realistic assessment was derived. It does not report evidence of significance in this manuscript; it describes improvement in HbA1c for some participants, including cases with improvement greater than 1 percentage point.	Yes. It focuses on mechanisms/contexts of change and self-efficacy (PAID-5) as a mediator of behaviors; it describes that the app supported self-management (in terms of experience and self-care practices), without quantifying “adherence” as a primary outcome in this article.	No formal measurement of QoL was identified in the retrieved extracts.	Engagement described qualitatively (for whom it “worked” and under what circumstances) rather than usage metrics or percentages.
12	Dsouza et al. (2024)	Qualitative study (semi-structured interviews)	10 patients with DM2 and 6 healthcare professionals	NOT APPLICABLE (explores mHealth influences and solutions for self-management)	NOT APPLICABLE	It does not report HbA1c as a result of the study (it is qualitative on barriers and drivers).	Addresses self-management behaviors as part of the context: physical activity and family support (among others). Example: “Everyone” reported doing daily physical activity (walking).	QoL measurement is not identified as an outcome.	Use/engagement with mHealth: 100 percent of the 30 participants did not use mHealth apps for diabetes; 86.8 percent did use the internet to find information (mainly Google and YouTube).

13	Feng et al. (2023)	Randomized controlled trial, 2 parallel groups	228 recruited; 225 followed up	Family eHealth intervention via WeChat (plus usual care) vs. usual care	1 year	Yes. Significant reduction: β = −0.69 ($p < 0.05$) for HbA1c in the intervention group. Range: not reported as a range; estimated effect (beta) reported.	Yes. Significant improvements in self-care (SDSCA): diet (β 0.34), exercise (β 0.46), glucose self-monitoring (β 0.42), and foot care (β 0.20), as well as an increase in overall family support behaviors (β 0.89) and in subdomains (e.g., diet support β 0.35; exercise β 0.32; foot care β 0.22; monitoring β 0.16).	No QoL measurements are identified in the retrieved extracts.	No satisfaction/engagement metrics (percentages of use, frequency, etc.) were identified in the extracts.
14	Georgsson y Staggers (2016)	Feasibility study: usability assessment with a multi-method approach	10 users (random sample from a larger clinical trial)	Usability evaluation of mHealth system for self-management of DM2 (tasks + think aloud, interview, questionnaire)	NOT APPLICABLE (usability evaluation session)	No (it is a usability assessment; it does not report HbA1c).	No (the focus is on the usability of self-driving features, not measured behavioral changes).	No.	Engagement/usability: 117 initial problems were identified, consolidated into 19 issues; average severity 2.47 (serious). They also describe the technological use of the sample (for example, daily internet use 70 percent).

15	Hall et al. (2018)	Mixed methods: focus groups + survey	Focus groups: 9 participants; Survey: 158 complete responses (out of 321 distributed)	NOT APPLICABLE (preferences regarding DM education and delivery formats)	NOT APPLICABLE	No.	Focuses on education preferences and support for self-management (not pre-post behavioral outcomes). Key finding: preference for 1:1 sessions (62.4 percent), followed by group (29.0 percent) and self-directed (8.6 percent).	QoL measurement is not identified as an outcome.	Engagement patterns as preference: participants wanted timely access to education as needed, with professional support (relevant for intervention design).
16	Hawkes et al. (2024)	Qualitative study, cross-sectional design (semi-structured interviews)	19 participants	Digital intervention “Healthy Living” (structured web education + self-management tools)	NOT APPLICABLE (cross-sectional interview; users had interacted with the program in the last 12 months)	No.	Yes, in qualitative terms: users reported that the platform supported self-management and generated perceived changes (motivation, habits, follow-up).	No formal measurement of QoL was identified as an outcome in the retrieved abstracts.	Engagement: There is a reported need for greater “interactivity” to sustain usage; users preferred support, feedback, and features that promote continuity.

17	Hermanns et al (2024)	Randomized, multicenter, open-label, parallel-group clinical trial	Estimated sample size: 225 (planned 251 with 10% dropout rate)	My Dose Coach (app for basal insulin titration + web portal) vs. standard titration without app	12 weeks	Yes, but as a protocol (without results): the primary outcome is HbA1c measured at the 12-week follow-up.	Yes, as secondary outcomes (not included in the protocol): self-management, empowerment, self-efficacy, satisfaction with treatment, and others.	Yes, as a secondary outcome: psychological well-being. No improvements are reported because it is protocol.	Includes usability assessment at the end of the study using a questionnaire. No percentages are reported as per protocol.
18	Horner et al. (2017)	Qualitative study (focus groups and interviews) within the framework of an RCT	46 in the intervention arm; 31 participated in qualitative research (67%)	"Text to Move": personalized text messages to increase physical activity (2 per day)	6 months	No.	Focuses on facilitators and barriers for a messaging program (not clinical outcomes).	No.	Engagement: Interviews describe automation and "repetition" of messages; only 67 percent responded to weekly "check-in" messages.
19	Hu et al. (2022)	Single-group pretest–posttest	30 participants	Asynchronous DSME program via WeChat: 2 videos/week	12 weeks of intervention; follow-up at 3 and 6 months	Yes. Potential efficacy: HbA1c decreased by 0.5 (95% CI: −0.8 to −0.2) and at 6 months by 0.4 (95% CI: −0.7 to −0.1).	Yes, behaviors/self-management reported by participants: changes in diet (more vegetables; reduction in carbohydrates), more physical activity, and greater confidence in managing diabetes.	No formal measurement of QoL is identified as an outcome in the retrieved abstracts.	Engagement/satisfaction percentages: retention rate 97 percent; video view rate 92 percent; average satisfaction 9.9/10.

20	Hu et al. (2024)	6-month RCT pilot study (baseline, 3-month, and 6-month measurements)	23 dyads (11 intervention, 12 control)	12-week educational intervention with videos (2/week) via WeChat or SMS; waiting list control	6 months (12-week intervention)	Yes, it was measured (0, 3, and 6 months). Significant intragroup change in intervention (FAMILY): −0.61 at 3 months and −1.23 at 6 months (p = 0.014). Control: −0.44 at 3 months and −0.88 at 6 months (p = 0.064). Non-significant difference in change between groups: −0.18 (0–3 m) and −0.35 (0–6 m), p = 0.611.	Yes (self-management). Significant intragroup improvement in FAMILY: +10.05 (0–6 m), p = 0.009; control showed a trend (+7.15; p = 0.059), but the difference in change between groups was not significant (+2.89; p = 0.590).	QoL measurements are not reported as an outcome in the trial (the main results describe HbA1c, weight, and self-management, as well as feasibility/acceptability metrics).	Retention (patients): 90.91% vs. 83.3% (p = 0.589). Retention (family/friends): 90.9% vs. 75% at 3 months (p = 0.313) and 90.9% vs. 83.3% at 6 months (p = 0.589). Average video viewing rate: 76.8% (SD 7%). Satisfaction (0–10): patients 9.4/10 and family members 10/10.
21	Jafar et al. (2023)	Experimental	66 (62 completed)	Health coaching through the "Guru Diabetes" app	3 months	Yes; no significant difference between groups (intervention vs. control)	Yes; self-care behaviors "improved" in the intervention group, but there was no significant difference between groups.	Yes; no significant change between groups.	Reports "high acceptance/satisfaction" with the intervention (no percentages in the available excerpt).

22	Jeffrey et al. (2019)	Qualitative (semi-structured telephone interviews)	30 (16 app users, 14 non-users)	Not applicable	Not applicable	No (qualitative)	Yes, qualitative: use of apps for self-monitoring (glucose, diet, activity), support for self-regulation; barriers (cost, complexity, low digital literacy, burden)	Yes, qualitative: perception of benefits in control/daily life, but not quantified.	Patterns: preference for simple tools integrated into routines; abandonment when perceived value does not compensate for effort/cost
23	Karimi et al. (2023)	Descriptive qualitative	60	EatSmart: web/mobile healthy eating program with modules and SMS	12 weeks	No	Yes, qualitative (nutrition): participants report greater awareness and dietary adjustments; need for personalization and interaction	Not quantitative in the available extract	63% “enjoyed” the program; 57% would recommend it. Patterns: not used due to lack of time/forgetfulness; requests for more interaction and personalization.
24	Koot et al. (2019)	Feasibility study, single arm (pre–post)	100	GlycoLeap: app + educational curriculum + coaching	24 weeks (6 months)	Yes; significant reduction: −1.3 pp (completers) and −1.1 pp (total), p<.001	Yes; significant improvements in self-care: more days of glucose self-monitoring (0.6 to 2.3 days/week), dietary improvements (fruits/vegetables ↑; fats ↓)	Not reported as QoL in the retrieved extracts	Satisfaction: overall rating “good” 51% and “very good” 23%; would recommend (53% probably, 26% definitely). Willingness to pay 21%.

25	Krall et al. (2023)	Multipass study: pilot without control group (cohort) + survey of clinicians	Phase 1: 32 patients; Phase 2: 60 clinicians	BDTM Diabetes Care App (mobile app for diabetes management support)	3 months (phase 1)	Yes; significant reduction, average 2.5±2.6 pp, p<.001	It does not measure classic "adherence"; it evaluates self-management facilitation (use of features, educational resources).	No QoL; yes, "diabetes distress" (significant reduction)	Usage/engagement: average 10 logins; 84% opened the app within 1 day. High satisfaction: easy to use 81.4%; attractive interface 85.2%; useful 81.5%; would recommend 85.1%.
26	Krall et al. (2024)	Feasibility cohort	36	Mobile app for insulin titration for pharmacological management of DM2	3 months	Yes; significant reduction 2.1±2.2 pp, p<0.001; 31% achieved HbA1c <7%	Treatment-related behavior: glucose titration and self-monitoring; 69.4% achieved individual fasting glucose target; mean time to target 28.8 days	No QoL; yes distress (improvement on all scales)	Satisfaction assessed using DMSRQ-SF (treatment experience); detailed results refer to table (no percentages in the extract)
27	Kuo et al. (2024)	Observational study (questionnaires at T0 and T1)	81	Exposure/use of a "personal health assistant" app after training	1 month	No (adoption/usage model)	Yes, focus on intention/use: "current usage behavior" and changes in averages over time; decrease in key variables during use	No	Engagement/use: the model explained 88.4% of the variance in intention to use and 82.7% of usage behavior; main predictors: performance expectancy, satisfaction, facilitating conditions

28	Lauffenburger et al (2021)	Qualitative (semi-structured interviews)	20	Not applicable	Not applicable	Not as an outcome (describes average baseline HbA1c of 7.8% in sample)	Sí, cualitativo sobre adherencia y apoyo: pacientes reportaron omisiones de medicación (65% omitió al menos 1 día; media 1.15 días) y describen “cues/routines” y barreras	No	Yes, qualitative on adherence and support: patients reported medication omissions (65% omitted at least 1 day; mean 1.15 days) and described cues/routines and barriers.
29	Leon et al (2021)	Qualitative process evaluation (interviews and focus groups) within a pragmatic trial	60 (administrators 2, nurses 33, patients 25)	StAR2D intervention via SMS (automated messages up to 4 times per week)	12 months	In the trial: glycemic control did not improve (evaluation process confirms this).	Yes, SMS intervention for behavioral change; evaluation process reports perceived usefulness for habits, but with heterogeneity of response (no quantitative magnitudes in the extract).	Not reported on the statement	Patterns: choice of frequency/message categories in the trial; preference for 2 messages/week; selection of categories (diet 86.1%, glucose control 83.5%, physical activity 74.8%)
30	Leong et al (2022)	Open-label randomized controlled trial (open-label RCT)	181 (90 control; 91 intervention)	Educational program on the LINE social network with videos (TMU-LOVE) + usual care	3 months	Yes; HbA1c (and other biomarkers) decreased in both groups, but with no significant difference between groups	Yes; improved attitude and self-care in the intervention group	Yes; improved QoL in the intervention group	Satisfaction/acceptance: 100% rated the program as “very useful” or “useful”

31	Lewinski et al. (2021)	Descriptive qualitative study	20	Use of multiple mHealth devices to support self-management in DM2 (e.g., fitness tracker, glucometer, scale)	6 months	Not evaluated as an intervention outcome. Reports average baseline HbA1c of 7.9 ± 1.15 as a sample characteristic (no change).	Qualitative study: explores facilitators/barriers to using multiple devices (Fitbit, iHealth glucometer, BodyTrace scale) to support self-management behaviors. Participants reported finding it "feasible" to facilitate engagement in self-management behaviors, with no quantitative metrics of adherence.	No formal measurement of quality of life reported.	Qualitative engagement/satisfaction: issues of feasibility and usability of multiple devices identified; experiences and perceptions described, not percentages of use.

32	Li et al. (2022)	Single-arm feasibility trial (pre-post)	15	DSMES/self-management support intervention led by community worker and enabled by mHealth	12 weeks	Yes, pre–post at 12 weeks. HbA1c: 6.91 ± 1.08 to 7.04 ± 1.22; change +0.13; p = 0.96. No significant reduction (range of "reduction": not applicable in this study, because the change was slightly upward).	Yes, operationalized as self-monitoring behaviors and goal achievement. They reported: glucose self-monitoring 80% "on most days"; physical activity self-monitoring 73% "on most days"; weight self-monitoring 93% "on most days"; dietary intake self-monitoring 33% "on most days." In addition, goal distribution: 72% achieved 75–100% of their physical activity goals, 44% achieved 75–100% of their dietary goals, and 52% achieved 75–100% of their weight goals.	Quality of life measurement is not reported as a primary outcome in the retrieved fragments (no QoL scale or reported change in QoL was identified in the cited results).	Satisfaction: CSQ-8 mean 29.53 (SD 2.91). Retention: 93% completed the study; 87.5% completed all modules. Usage patterns: consistent use during the first 2 weeks, then decline over time; average monthly visits 3.75 (SD 2.33). Barriers to engagement: lack of motivation, lack of time, technical problems, forgetfulness.

33	Liu y Yu, 2024	Participatory design and usability study (heuristic evaluation + usability testing)	6 experts (heuristic) + 28 older adults (usability)	Development and refinement of app for DM2 in older adults (SugarShift)	Not applicable	No. The work focuses on design, heuristic evaluation, and usability; it does not report HbA1c as an outcome variable.	Not as a clinical outcome. It addresses requirements/functionalities for self-care (e.g., recording and consultation), but without longitudinal clinical or behavioral adherence results.	No measurement of quality of life or clinical improvements is identified; the focus is on usability and interface issues for older adults.	Usability/satisfaction: SUS in patient group (n = 28) mean 72.41 (SD 5.2), median 72.5, range 65–85; interpreted as “good” overall. In addition, heuristic evaluation identified 126 problems and 179 heuristic violations, indicating areas of friction that may affect engagement.
34	Maharaj et al. (2021)	Randomized controlled feasibility trial	58	Comparison of two commercial self-management apps (Glucose Buddy vs. mySugr)	2 weeks	HbA1c was recorded at baseline (range 5.3 to 15.4) from health records, but no reduction was reported as the primary outcome.	Self-care behaviors (e.g., glucose monitoring) were measured. Finding of relationship between glucose monitoring and time spent on app (positive correlation; association with use reported).	No QoL measurement reported.	Low-moderate engagement: median days of use 4.0 (Glucose Buddy) and 6.5 (mySugr); 0.75 logins/day; average uMARS 3.37. Barriers reported: apps perceived as demanding/complicated.

35	Mahoney et al. (2022)	Randomized controlled trial (parallel)	58	Combined diabetes self-management app with 4 mm pen needle vs. Control	8 weeks	HbA1c is not reported as an efficacy outcome; focus is on glycemic variability (e.g., MAGE) and PROs.	Adherence measured with ARMS-D: medication adherence improved in the control group (P=0.001); no significant differences between groups in other PROs.	No formal measurement of overall QoL reported	Engagement: “high variability” in app use (heterogeneous pattern). Satisfaction: app satisfaction scores are reported (scale 1 to 5; higher scores reflect greater satisfaction).

36	Mash et al., 2022	Mixed methods (convergent) for implementation evaluation	8,158 users connected (4,577 listened to messages)	GREAT4Diabetes WhatsApp chatbot with self-care education (messages/audio)	5 months	They do not evaluate HbA1c as a clinical outcome (article on implementation/scale and use of a WhatsApp chatbot; coverage and flow metrics). Therefore: no significant reduction or HbA1c ranges reported.	They report self-reported changes at the end of the program: "I improved adherence to medication" 44% (182/414). They do not present this as a pre-post comparison with a significance test; it is descriptive.	Does not report quality of life results.	Usefulness/satisfaction per message: more than 90% of those who listened rated the messages as "useful" (e.g., 93.9%–99.1% depending on the message). Use/abandonment: 8,158 "reached" the chatbot; 57.83% responded to T&C; 5.7% asked to stop the chatbot, and 62.4% of those did so within the first 24 hours. Significant drop-off: only 12.56% (575/4577) of those who received message 1 received the final message 16. They also compare completeness by recruitment channel (TV 4.53% vs. radio 12% vs. recruited by healthcare personnel 16.47%).

37	Mathiesen et al., 2017	Qualitative study (patient perspectives)	12	Experiences/perspectives on digital interventions for diabetes management in vulnerable populations	Workshops + interviews (3 workshops lasting 3 hours)	Not as a pre–post outcome. Reports HbA1c only as a sample characteristic: mean 76 mmol/mol (range 54–103).	Yes, but from a qualitative (not quantitative) perspective. Concludes that "diabetes distress" may act as an intermediate mechanism leading to non-adherence to digital interventions, with a preference for human interaction.	Not measured on a scale. Contextually mentioned that complications and disease are associated with a decrease in QoL. It does not report "improvements" because it is not an effectiveness study.	It does not report satisfaction/usage percentages. It identifies issues that explain low engagement or rejection: "experiencing digital alienation" (loss of freedom when technology invades the private sphere) and "missing the human touch" (preference for human contact), among others.
38	Mellergård et al (2021)	Qualitative study	22	Development/evaluation of web-based self-affirmation support to motivate lifestyle changes	Not applicable	Does not report HbA1c as an outcome. Qualitative study of perceptions.	Explores perceptions of self-care and support needs (does not measure quantitative adherence).	Does not report formal measurement of QoL	Qualitative engagement/satisfaction: reports perceptions, barriers, and facilitators regarding web intervention; does not report usage percentages.

39	Mitchell et al. (2020)	Mixed methods study embedded in a trial	158 (virtual world arm; total trial 309)	Education and support for self-management in the virtual world (weekly group sessions)	8 weekly sessions (8 weeks)	Reports mean baseline HbA1c 10.0 (SD 2.0), but the shared document focuses primarily on presence/engagement metrics rather than clinical changes.	Focused on participation in education program/group visits in virtual world; available PDF does not present quantitative results of typical behavioral adherence (medication/diet), but rather experience/participation.	No QoL measurement is identified in the available excerpt.	Engagement: evaluates "presence" and experience in the virtual world through domains (e.g., "involvement," "fidelity"); averages are reported by item, not patterns such as daily logins.
40	Mueller et al. (2024)	Observational study (baseline analysis)	101	Routine use of adherence support app (MyTherapy) + baseline measurement of disease knowledge/awareness	3 months	HbA1c was collected as self-reported clinical data: 75.1% reported HbA1c within 12 months. This is not a reduction study (no intervention).	Does not report changes in adherence; study describes a cohort and baseline measurements (includes comorbidities and reported measures).	Quality of life measured with EQ-5D: average index 0.84 and EQ-VAS 74.21.	Satisfaction: reports average DTSQ (treatment satisfaction) 27.26. Daily frequency usage patterns are not detailed in the available extract.

41	Mueller et al. (2022)	Cross-sectional, single-arm study (feasibility of a fully digital workflow)	n=29 eligible; n=28 completed	Digital workflow to capture PROs using an app (my ePRO) with authentication/2D code on the drug packaging	6 months	Yes, reported the "last glycosylated hemoglobin value" as descriptive (no intervention, no comparison, no reduction). Distribution (%): missing 7; <6.0: 38; 6.0–6.5: 10; 6.6–7.0: 14; 7.1–7.5: 14; 7.6–8.0: 0; 8.1–8.5: 10; 8.6–9.0: 3; >9.0: 3.	Assessed self-care with SDSCA: total average 3.5 days (SD 1.3) out of 7 days. Also reported self-reported events: hyperglycemia or hypoglycemia in 69% (20/29) and 24% (7/29), respectively.	Yes. EQ-5D-5L: "best possible health status" reported by 64% (18/28). Does not report pre-post change (observational design).	Yes (satisfaction with treatment). DTSQ: 83% (24/29) "satisfied to extremely satisfied" with current treatment. Does not report usage metrics such as logs (it was not an engagement trial).

42	Muller et al. (2017)	Randomized controlled trial (2 arms)	n=1045 completed the trial	Web-based information on physical activity for DM2: interactive format vs plain text	Single session; baseline and immediate post-intervention assessment	HbA1c is not reported as a study outcome.	Behaviors assessed indirectly: web-based intervention to promote physical activity. Results: health literacy outcomes (including attitudes and intentions to engage in physical activity) improved significantly after the intervention in both groups, but the interactive version did not produce better results than the plain text version.	Does not report QoL measurements.	Yes (engagement and satisfaction). Use: the "plain text" group reviewed significantly more sections of the material (mean difference −0.47; 95% CI −0.64 to −0.30; P<.001), without translating into better outcomes. Knowledge: the interactive group acquired more knowledge about diabetes (mean difference 0.80; 95% CI 0.65–0.94; P<.001). Satisfaction: reports "high satisfaction" and willingness to recommend the site, without percentages in the summary.

43	Newhouse et al. (2025)	Qualitative study (semi-structured interviews) nested within an RCT	n=43 interviewees	NOT APPLICABLE (qualitative study on the experience with the SuMMiT-D digital intervention)	Interviews in week 4 and week 52 of follow-up	Does not report HbA1c (qualitative process evaluation).	Yes, central focus: adherence to oral medication through SMS intervention (SuMMiT-D). Qualitative findings: two general themes; messages provided “holistic” benefits and supported cognitions, beliefs, and behaviors necessary for self-management; perceived value was variable and context-dependent (routines, lifestyle disruptions, understanding of DM2, relationships, attitudes).	Does not report QoL measurement.	Yes, but qualitative (no percentages). Engagement described as context-dependent and subject to subjective assessment of usefulness over time (no quantified usage patterns).

44	Öberg et al. (2018)	Qualitative study with individual interviews	n=11 (diabetes specialist nurses)	NOT APPLICABLE (explores perceptions of eHealth services)	Data collection Feb–Oct 2016; interviews 40–80 min (median 60)	Does not report HbA1c (qualitative study).	Addresses self-care/self-management as a topic: perceptions of eHealth for self-management support. Findings: mixed feelings; potential benefits such as greater involvement, empowerment, and security; concerns about ambivalence and uncertainty; need for training in digital skills and more person-centered solutions.	Does not report quality of life measurement.	Qualitative satisfaction/engagement (no percentages): acceptance conditioned by perceived benefits vs. concerns; recommendation for training and adaptation to individual needs.

45	Orozco-Beltrán et al. (2022)	Multicenter, randomized (2:1) intervention vs. control study; subsequent reassignment of use at 52 weeks	n=98 recruited; n=89 evaluable; n=50 completed evaluation at week 24	DeMpower app with connected devices (scale, glucometer, BP, activity bracelet) and wireless data transmission to healthcare team, with training	52-week follow-up (primary endpoint: week 24)	Yes. Significant reduction in HbA1c at week 24: −0.81 (SD 0.89) vs −0.15 (SD 1.03); P=.03. Also reports proportion with goals: HbA1c ≤7.5%: 64% vs. 24% (P=.02); HbA1c ≤8%: 85% vs. 53% (P=.02). The target "HbA1c ≤7.5% with reduction ≥0.5%" showed a trend: 46% vs. 18% (P=.07).	Yes. Adherence: MARS-5 showed similar adherence in both groups at week 24, with a "positive trend" in the intervention group. (The abstract also mentions trends toward improvement in adherence).	It is not reported as a primary outcome in the main text; references to "quality of life" appear more as discussion/background.	Yes (but with limited data in the main text). Satisfaction: DTSQs and IEXPAC were "positive" in both groups, with no significant differences between groups (see supplementary table). Engagement/patterns: the design considered subgroups of app use (long-term vs. short-term use), but the main body does not show specific retention/use percentages; integration with devices and data transmission are described.
46	Piette et al. (2024)	Online survey (latent class analysis)	n=633 adults with T2DM	NOT APPLICABLE (observational study on use/engagement with digital health technologies	3 months	Did not measure HbA1c as a clinical outcome; reported self-reported HbA1c by ranges (distribution of participants).	Addressed adoption/use of BEAMS technology (usage behavior), with adoption profiles (e.g., "any use" and subgroups).	No quality of life measurement was identified in the retrieved extract.	Engagement focused on adoption: "adopters" (use at any time) and three subgroups (21%, 42%, 37%) with different combinations of use.

47	Poduval et al. (2020)	Mixed, naturalistic "in the wild" study (real-world usage data + questionnaires + interviews)	n=791 registered; n=74 completed the program	Structured web-based self-management education program: Healthy Living for People with Type 2 Diabetes: Starting Out (HDSO)	Pre/post program assessment: week 1 to week 4	No: states that clinical data could not be collected (no access to records).	Yes, but as "psychological outcomes/self-efficacy and distress" (related to self-care): improvement in self-efficacy (median change 2.5; P=.001) and reduction in distress (median change 6.0; P=.001).	No explicit measurement of quality of life was identified	Low engagement (in real context): of 791 registered, 74 completed (9.0%).
48	Prinjha et al. (2020)	Qualitative study (focus groups and semi-structured interviews)	n=67	mHealth intervention with text messaging component to improve medication adherence (in a British population of South Asian origin)	6 months	No HbA1c reported (qualitative study on SMS and adherence).	Yes: explores medication adherence (perceptions, barriers, and facilitators) in messaging intervention.	Not applicable (quality of life measurement not retrieved).	No satisfaction/usage percentages identified in the abstract; focus is qualitative (acceptance and meaning of SMS).
49	Rossmann et al. (2019)	2 qualitative studies (interviews in Singapore; focus groups in Germany)	Study 1: n=21; Study 2: n=16	NOT APPLICABLE (explores appropriation/use of mHealth for self-management)	Study 1: Dec 2015–Sep 2016 (interviews ~1 hr); Study 2: Jun–Aug 2017	No (qualitative study on mHealth appropriation).	Yes (qualitative): describes how people "appropriate" mHealth for self-management; findings focused on patterns/roles of use rather than clinical changes.	Not identified.	Engagement described qualitatively (patterns of appropriation and use); no percentages in the retrieved extract.

50	Spierling-Bagic et al. (2023)	Process evaluation (RE-AIM) within a study with 3 intervention groups	n=310 enrolled (out of n=571 eligible evaluated)	mHealth intervention with device integration, alerts, and follow-up; includes arm with telephone Health Coach (DD-Me-Telephonic-HC)	Follow-up reported at 6 and 12 months (retention 92% and 90%, respectively); enrollment Oct 2017–Mar 2020	HbA1c appears as an eligibility criterion (≥8.0%) and as a baseline clinical variable to compare eligible vs. enrolled participants. However, this article is a process evaluation (RE-AIM) and notes that clinical effectiveness analyses (including improvement in control) are "ongoing"; it does not report changes, significance, or ranges of HbA1c reduction.	It does not report "significant improvements" in adherence as a clinical/patient-reported outcome. It does report monitoring/engagement related to adherence: alerts for "no pill box openings" (indirect indicator) and coach coverage: in the group with calls, 98% of calls discussed medication adherence.	It does not report quality of life outcomes.	Engagement/retention: 92% remained "engaged" at 6 months and 90% at 12 months (completing surveys or lab tests). Satisfaction/perception: mean score 28.4/32; 81% said they read the texts; 86% said they liked receiving calls/texts; 93% said the program helped them manage their diabetes; 99% said they would recommend it. Engagement metric: overall EMA response rate ~50.7% (and differences between arms).

51	Sze et al. (2023)	Pilot study, single-group pretest–posttest design	33	StepAdd mHealth system with app and devices (pedometer, BP monitor, scale), with monthly support meetings with community pharmacist	2 weeks baseline + 12 weeks intervention	Yes. Significant reduction: HbA1c from 7.91±0.88 to 7.12±0.59; difference −0.79±0.83; P=0.0001.	Yes (behaviors/self-care): improved physical activity (achievement of step goal) and improved scores in diet, exercise, foot care, self-efficacy, and self-regulation.	No direct measurement of quality of life was identified in the results retrieved (self-care domains and clinical variables are reported).	Satisfaction/use (percentages): high perceived acceptance (items on usefulness and ease of use). Example: "very useful" 83.9%, 'useful' 16.1%; "very easy to use" 61.3%, "easy" 35.5%. Patterns: low rate of daily goal achievement (12.8%) but with improvements in steps and self-monitoring.
52	Tang et al. (2025)	Randomized, parallel, open-label, superiority, multicenter factorial clinical trial (4 groups)	273	Dnurse (app + smart glucometer) and/or telephone follow-up, compared with usual care	6 months	Yes (as HbA1c goal achievement). Significant improvement in HbA1c attainment rate: RR 1.74; 95% CI 1.19–2.54; P=0.005; and no significant differences in HbA1c level.	Yes. Reports improvement in self-monitoring of blood glucose (SMBG) compliance: at 3 months 91.0% vs. 59.7% and at 6 months 86.9% vs. 57.0% (intervention vs. control).	Not identified in the abstract retrieved.	High satisfaction (means >4.5 in survey). Retention: 98.3% completed 6-month follow-up. Detailed usage patterns (logins/time) not retrieved in the abstract.

53	Torbjørnsen et al (2019)	Descriptive qualitative study (semi-structured individual interviews)	24	Use of a “digital diabetes diary” app (mHealth) in people with T2DM	12 months	No (qualitative on acceptability).	Indirect: explores app-supported self-management experiences; does not report quantitative changes in adherence.	Not identified.	Engagement and acceptability described qualitatively (experiences/factors that facilitate or hinder). No percentages in the abstract.
54	Torbjørnsen et al. (2018)	Randomized clinical trial with three arms	101	Diabetes diary app alone, app + telephone counseling, versus control	12 months	Yes. Reported HbA1c (medians): for example, 7.8 to 7.6 (intervention). No test of significance of the change was recovered in the extract.	Includes self-management and empowerment outcomes (heiQ and others), and compares high vs. low frequency of use; specific adherence values (e.g., medication) were not retrieved here.	Not identified as a quality of life measure; outcome scales related to self-management/psychosocial impact are used.	Engagement with usage data: high-frequency users 48%, defined as use on more than 18 days/month and more than 7 pulses/day.
55	Tyagi et al. (2024)	Pilot feasibility study (one-group, pre- and post-intervention)	37 recruited; 33 completed	PTEC HAT program: self-testing of HbA1c at home with kit + telemonitoring and teleconsultations	Comparison of indicators “during/at the end of the intervention” versus 6 months before	Yes. Pre/post HbA1c described as comparable (no significant reduction in the summary).	No direct measures of adherence were recovered; the focus is on the feasibility/acceptability of home self-testing and telemonitoring.	Not identified.	Satisfaction/acceptability: 81.3% considered it beneficial; barriers: Bluetooth 43.7%, too many steps 28.1%; willingness to pay 22%.

56	Whittemore et al. (2025)	Pilot study, within-subject (pre-test–post-test)	23	Multilevel MAP intervention to increase access/use of patient portal, with nursing communication (messages/calls)	6 months (3 months intensive + 3 months weekly)	Measurement. Mean: 8.31 (baseline), 8.09 (3 m), 8.23 (6 m). No significant change; slight decrease at 3 m and return to baseline at 6 m.	Self-care behaviors (SDSCA: diet, exercise, glucose monitoring, foot care, medication). No significant change in self-care behaviors.	No formal measurement of quality of life reported. (Psychosocial variables such as PAID diabetes distress are reported, not QoL).	High satisfaction: 4.03 (3 m) and 4.14 (6 m) on a scale of 1 to 5; 100% would recommend the program. 100% would continue to use the portal and consider it useful. Engagement: 100% activated their account and logged in during the first month; 3.16 logins/week (0-3 months) and 1.45 logins/week (3-6 months). 96% (20/21) met “2 or more logins/month” in 0-3 months and 76% (16/21) in 3-6 months.
57	Whittemore et al. (2020)	Formative research and intervention development/implementation (formative research, prototyping-validation, implementation phases)	Not applicable	DSME + mHealth program (pictographic messages, video novels, and community support)	7 weekly group sessions (plus weekly support messaging)	Not applicable: development/design article (does not report clinical results).	Not applicable: describes the development of an HAPA-based mHealth intervention and adaptation process; does not report adherence or behavior results.	Does not report quality of life measurement.	Does not report satisfaction/engagement as results (methodological development approach).

58	Wieland et al. (2024)	Randomized clinical trial	451	Culturally adapted digital storytelling intervention for DM2 self-care	3 months	Measurement. Baseline change at 3 months: intervention 9.1 to 8.4; control 9.4 to 8.8; significant difference in adjusted model (P=0.04).	Reports that no significant differences were observed in secondary outcomes (including cardiometabolic and psychosocial measures evaluated).	Does not report formal quality of life measurement. (Psychosocial variables were evaluated, but the article does not report QoL as a primary outcome).	Engagement/retention: 86% completed 3-month follow-up; feasibility 100% (met recruitment and retention goals). Acceptability (percentages): 90% felt that the videos were for people like them; 80% indicated that they learned something new; 97% did not feel uncomfortable; 87% wanted to see more stories; 94% felt motivated to take care of their diabetes. Immersion/identification experience: 68% felt immersed in the story; 64% felt intense emotions; 68% identified with the character.

59	Ye et al. (2020)	Descriptive qualitative study (semi-structured interviews)	17	NOT APPLICABLE (explores perceptions of remote glucose management using apps by adult children)	Use of apps for more than 6 months (inclusion criterion)	Does not report objective HbA1c measurement as an outcome (qualitative study). However, narrative results mention perceived improvement in glucose.	Addresses behaviors and adherence from experience: the app shows adherence rate, and participants describe greater awareness/action (e.g., exercise after eating, regular glycemic control, diet). An increase in adherence from 50% to 70% and achievement of 50% to 80% of glycemic control goals are reported (according to study narratives).	No formal measurement of quality of life is reported.	No classic satisfaction percentages are reported; however, acceptability/use by topic is reported: valued features include activity tracking, medication, and diet; participants report that the app is “easy to use” and provides monitoring and a sense of control.

60	Yang et al. (2020)	Cluster randomized clinical trial, with clinics as the randomization unit	254 (153 intervention; 101 control)	Glucose monitoring and feedback system via mobile phone, integrated into primary care (compared with usual care)	3 months	Measurement. At 3 months: adjusted mean difference in change between groups in HbA1c of −0.30% (95% CI −0.50 to −0.11); 33.8% achieved HbA1c below 7% in intervention vs. 24% in control.	Adherence and behaviors: MMAS-6 increased more in the intervention (adjusted difference 0.31); the “motivation” subcomponent also increased more (difference 0.23). Self-monitoring behavior: defines “good SMBG adherence” as an average daily count greater than or equal to 1; reports differences in compliance (better reduction with good adherence).	Does not report formal measurement of quality of life.	Satisfaction: DTSQs increased more in the intervention (adjusted difference 2.21 points). An increase in satisfaction is also reported only in the intervention and an increase in the total MMAS-6 score. (The article also reports analysis by SMBG compliance and its relationship to HbA1c reduction).

61	Yingling et al. (2019)	Exploratory study with digital tool testing and semi-structured interviews	21	Testing of digital health tools for self-management: iPad, Fitbit, Libre Pro CGM, and MyFitnessPal app	1 week	Does not measure HbA1c as an outcome of change (short study); reports mean baseline self-reported HbA1c of 7.4 (SD 1.8).	Behaviors: dietary log and physical activity as indicators of self-management. 52% logged at least one meal; 33% logged 4 days or more. Physical activity data was logged 7.1 days on average; average of 7,335 steps/day.	Does not report formal measurement of quality of life.	Engagement and preferences: patterns of diet diary use (52% some meals; 33% 4 days or more) and tracker use (7.1 days; 7,335 steps/day). Perception/acceptability: the focus group suggests a more positive experience with RT-CGM (continuous monitoring) and the opportunity for real-time support and motivation to improve behaviors.
62	Yoon (Sungwon) et al. (2024)	Qualitative study (semi-structured interviews)	33	NOT APPLICABLE (explores acceptability and preferences regarding an app based on motivational interviewing; not an intervention trial)	Not applicable	Did not measure HbA1c (qualitative study; acceptability/preferences). Does not report significant reduction.	Explores barriers and facilitators for self-management and motivation (interviews; N=33). Suggests that MI (motivational interviewing) can support behavioral changes; some preferred self-management at their own pace.	Did not assess quality of life with instrument; appears as context/motivation (without quantitative measures).	High acceptability of MI via app, but doubts due to lack of “human touch.” Preference for hybrid model (automated + human coaching). Suggested features: milestone visualization, gamified challenges, incremental rewards, goal-specific multimedia resources, interactive and empathetic conversation.

63	Yoon (Sungwon) et al. (2022)	Qualitative study (semi-structured interviews)	29	NOT APPLICABLE (identifies goals, barriers, and desired functionalities for an mHealth app)	Not applicable	Did not measure HbA1c (qualitative study). Does not report significant reduction.	Addresses self-care and behaviors: goals focused on better glycemic control and medication reduction; barriers: competing priorities, limited healthy food options at work, and lack of personalized advice. Results (qualitative; n=29): desired functionalities to improve self-care behaviors include quantifiable goals, personalized nudges based on recorded data, integrated resources from credible sources, social support within the app (virtual interaction with peers and health professionals), novel technology-driven data recording, and user-defined nudges.	Did not measure QoL; mentioned as expected long-term gain (without measurements).	Does not report satisfaction percentages. Identifies determinants of engagement by design: quantifiable goals, personalized nudges, in-app social support, and credible resources (user preferences; n=29).

64	Shen et al. (2025)	Qualitative study (semi-structured interviews)	15	NOT APPLICABLE (analyzes facilitators/barriers to continued use of apps; actual users of the MMC app)	Previous use of the app: more than 1 month. (reported range: 1 month to 3 years)	Yes, as a clinical characteristic (not as an intervention outcome). HbA1c available in the last 3 months for 8/15 participants: 5.5%, 6.1%, 6.4%, 6.8%, 6.9%, 7.5%, 10.3%, 11.2%; 7/15 had no recent measurement. Does not evaluate changes or significance.	Focuses on adherence to the use of diabetes apps (continuous use): internal facilitators (concern for health, symptoms, self-efficacy) and external facilitators (feedback, convenience, family/professional support). Barriers: technical/usability problems, unreliable or non-personalized content, and lack of human support. App with modules for recording (glucose, BP), education, diet, consultation, and medication (N=15).	Did not measure QoL with an instrument; discussed indirectly (better self-management/prevention of complications).	Usage patterns: duration of app use between 1 month and 3 years; some reported decreased use over time. The study details facilitators and barriers to continuity (sustained engagement), without satisfaction percentages.

65	Zamanillo-Campos et al. (2022)	Qualitative study (focus groups)	34	NOT APPLICABLE (collects opinions for the design of DiabeText; intervention still in design phase)	Not applicable	Did not measure HbA1c (qualitative study). Does not report significant reduction.	Main focus: adherence to oral antidiabetic drugs (design of SMS intervention "DiabeText"). Results (qualitative; N=34): patients identified barriers to self-care (information/support on diet and physical activity) and to taking medication (eating out, traveling, polypharmacy, dispensing at the pharmacy). They considered that medication support was not the most urgent need for everyone.	QoL was not measured; it is inferred as an expected benefit of better self-care (without measurements).	High acceptability and perceived usefulness if the content addresses barriers and has specific characteristics: short, clear messages and personalized information. No percentages reported.

66	Zhang et al. (2023)	Cross-sectional study (survey, 2021)	559	NOT APPLICABLE (assesses willingness to use mHealth apps and its association with complications)	Not applicable	Did not measure HbA1c (cross-sectional survey on intention to use apps).	Assesses intention to use disease management applications (DMAs) as a proxy for future behavior; models relationships: perceived ease of use (β=0.511, P<0.001) and e-health literacy (β=0.115, P<0.05) predict perceived usefulness; digital literacy predicts ease of use (β=0.659, P<0.001); usefulness (β=0.137, P<0.001) and attitude (β=0.825, P<0.001) predict intention to use.	Did not measure QoL; mentioned in background (no evaluation).	Measures attitude and intention (Likert scale 1–5), not satisfaction with actual use. Intention means: ITU1 3.49(1.21), ITU2 3.36(1.17), ITU3 3.46(1.21). The model explained 40.6% of the variance in ease of use, 35.2% in perceived usefulness, 68.7% in attitude, and 87.2% in intention. It does not describe usage patterns (logins/frequency) because it is not a study of actual use (n=559).
