# Supplementary material for: User experiences and perceptions on the use of digital health technologies in the management of type 2 diabetes: an integrative systematic review
Source: Front Clin Diabetes Healthc. 2026 Feb 3;7:1750055. doi: 10.3389/fcdhc.2026.1750055 (PMC12909238; doi:10.3389/fcdhc.2026.1750055)
Supplement: Supplementary file 2 [file DataSheet2.pdf]

Number	Database	DOI	Author (year)	Gender	Title	Country	Type of study	Level of Evidence	Quality of evidence	Experiences	Perceptions	Barriers	Facilitators
1	Scopus	10.2196/jmir.8439	Pal et al. (2018)	Female	Digital health interventions for adults with type 2 diabetes: Qualitative study of patient perspectives on diabetes self-management education and support	United Kingdom	Qualitative	III	High quality			Barriers to the use of mobile applications include lack of knowledge about potential benefits, inconvenience, fear of stigma, and lack of trust in technology. (Kingshuk, 2018)	Participants considered access to their electronic medical records and features such as appointment booking to be important facilitators, as they increased their engagement and facilitated disease management. In addition, the ability to receive regular reminders and emails was also seen as a factor that could improve participation in digital interventions. (Kingshuk, 2018)

2	Scopus	10.2196/16692	Baptista et al. (2020)	Female	User experiences with a type 2 diabetes coaching app: Qualitative study	Australia and New Zealand	Qualitative	III	High quality	The experiences of the study participants showed that their needs, preferences, and expectations regarding the use of the app varied according to their diabetes self-care styles. Participants with a more self-directed self-care style were more proactive and committed to using the app, while others showed less engagement. In addition, factors such as individual characteristics and the suitability of the app influenced their level of commitment and satisfaction. Overall, participants rated the app as useful.	Participants' perceptions of mobile app use varied and depended on their self-care styles. Some valued the app's usefulness in monitoring and managing their disease, but also expressed that personalization and additional support could improve their experience and motivation. In addition, participants with a more self-directed self-care style were more proactive and committed to using the app, while others showed less engagement, influenced by their individual expectations and needs.	The barriers faced by participants included a lack of personalization of the applications, difficulties in integrating them into their daily routines, and the perception that the apps were not always relevant or easy to use for their individual needs. In addition, some expressed that motivation and commitment could diminish over time, and that the lack of additional support limited their effectiveness.	Facilitators for participants included the perceived usefulness of the app for monitoring and managing their diabetes, as well as access to resources and technical support, such as the availability of a program coordinator to assist with technical difficulties and the integration of features such as reminders and feedback that motivated its use.

3	Scopus	10.2196/17038	Baptista et al., (2020)	Female	Acceptability of an Embodied Conversational Agent for Type 2 Diabetes Self-Management Education and Support via a Smartphone App: Mixed Methods Study	Australia and New Zealand	Mixed	II	Good quality	The experiences of the study participants indicated that the mobile application and the embedded conversational agent (Laura) were generally accepted and considered useful for managing type 2 diabetes. Participants valued the personalized interaction, education, and motivational support provided by Laura, and found that the app facilitated self-management of their disease in their daily lives. However, some expressed that interaction with the agent could be limited or that they would prefer more variety in conversations and functions.	Participants' perceptions of using the mobile app with a conversational agent for managing type 2 diabetes were generally positive. Participants valued the personalized interaction, ease of use, and usefulness of receiving education, feedback, and motivational support through Laura. Some mentioned that the app helped them stay motivated and improve their self-management, although there were also comments about the need for greater variety in conversations and features.		Facilitators for participants in using the mobile app included personalized interaction with the conversational agent Laura, who provided education, feedback, and motivational support tailored to their individual goals and conditions. In addition, the ease of use of the technology, such as voice recognition and natural conversations, facilitated acceptance and participation in the program.

4	Scopus	10.2196/17537	Batch et al. (2021)	Male	General Behavioral Engagement and Changes in Clinical and Cognitive Outcomes of Patients with Type 2 Diabetes Using the Time2Focus Mobile App for Diabetes Education: Pilot Evaluation	United States	Qualitative	II	Good quality	Participants in the study reported high satisfaction with the mobile app, rating it an average of 4.56 out of 5 and considering it relevant to their daily lives. They also indicated that the app helped them better manage their diabetes and that they would recommend its use to others, highlighting that the app was informative, motivating, and helped them stay accountable.			Participants highlighted that the app's design, which allows users to progress at their own pace, is based on real-world situations and focuses on skill acquisition and confidence building, thus facilitating its use and acceptance. In addition, they appreciated that the app was informative, educational, motivating, and helped them stay accountable, which encouraged their commitment to managing the disease.

5	Pubmed	10.1177/20552076221147109	Blythin et al. (2023)	Female	Can digital health apps provide patients with support to promote structured diabetes education and ongoing self-management? A real-world evaluation of myDiabetes usage	United Kingdom	Qualitative	II	Good quality	The experiences of study participants indicate that 89% of users who activated the app took steps to self-manage their diabetes, such as watching structured educational videos. In addition, 54.3% of users recorded clinical entries, such as HbA1c, blood sugar levels, and weight, demonstrating that the app facilitated monitoring for both patients and clinicians.		The article mentions that the specific barriers to using mobile applications are not known in detail, but suggests that factors such as failure to receive the access link, lack of technological skills, lack of internet access, or possible lack of interest in participating may have influenced the non-activation of the app. Furthermore, it indicates that neither age nor gender appeared to be significant barriers to using the app, although participation in activities such as watching educational videos did not show statistically significant differences between age groups.	The article mentions that during the COVID-19 pandemic, the use of digital platforms such as the myDiabetes app increased significantly, facilitating access to diabetes education and self-management, especially for those who had difficulty attending in-person programs. In addition, the availability of structured educational content and the ability to record clinical data in the app also facilitated user participation and engagement.

6	Scopus	10.1136/bmjdrc-2019-000981	Boels et al. (2019)	Female	Effectiveness of diabetes self-management education and support via a smartphone application in insulin-treated patients with type 2 diabetes: Results of a randomized controlled trial (TRIGGER study)	Netherlands	Quantitative	I	High quality			The article mentions that one of the limitations was that most participants had a high level of education and that the study mainly included secondary care patients, which may limit the generalizability to people with lower socioeconomic or educational levels. In addition, the use of other diabetes apps was not monitored, which could have biased the results toward nullity. However, no specific barriers perceived by participants in relation to the use of the app are reported. (Boels, 2019)	

7	Pubmed	10.2196/31451	Bults et al. (2022)	Male	Barriers and Drivers Regarding the Use of Mobile Health Apps Among Patients With Type 2 Diabetes Mellitus in the Netherlands: Explanatory Sequential Design Study	Netherlands	Qualitative	III	Good quality	The experiences of the participants in the article indicate that those who use mobile applications for managing type 2 diabetes believe that these tools can help them improve their personal health and well-being, allowing for more frequent monitoring and immediate reaction to changes in their glucose levels. However, they also mention that the time and energy required to record and understand the data in the apps represent barriers, and some are unsure whether using apps actually improves their well-being or reduces health problems.	Participants' perceptions indicate that many believe these tools can facilitate better glucose level control and promote an active and healthy lifestyle. However, there are also doubts about whether using apps actually improves personal well-being or helps reduce health problems, and some consider that the effort required to record data can be a barrier. Perceived ease of use is generally positive, with most considering the apps to be easy to use.	The main barriers mentioned by participants in the article were the time and energy required to record and understand data in the applications, which can hinder their use and perceived usefulness.	The facilitators mentioned by participants in the article include that most (95.1%) had a smartphone or computer with internet access and sufficient knowledge to use the apps. In addition, some patients had support from healthcare professionals and others had someone to help them in case of problems. However, they also pointed out that the lack of funding or reimbursement from insurance companies was a barrier, as many had to pay for the apps out of their own pockets.

8	Scopus	10.2196/10324	Burford et al. (2019)	Female	Small data and its visualization for diabetes self-management: Qualitative study	Australia	Mixed	III	Good quality	The experiences of participants in the study on the use of mobile applications for managing type 2 diabetes were generally positive. Participants were able to collect, view, and understand their personal health data, such as glucose levels, weight, and blood pressure, using graphs and charts on tablets. This allowed them to interpret the impact of their decisions and behaviors, empowering them to improve their health and make changes to their daily routine.	Participants' perceptions of using mobile applications to manage their type 2 diabetes were positive, highlighting that the applications allowed them to record, view, and interpret their personal health data, such as glucose levels, weight, and blood pressure. This helped them better understand the impact of their decisions and behaviors, empowering them to make changes to their daily routine and improve their health.	The article mentions that, although mobile applications offer advantages for managing type 2 diabetes, some participants faced difficulties in integrating, viewing, and interpreting the personal data collected with these technologies. The management, visualization, and understanding of health data, especially on devices with small screens such as smartphones, represent significant barriers to their usefulness and effective use.	Facilitators for participants in the use of mobile applications for managing type 2 diabetes included the ability to record, view, and understand their personal health data through graphs and charts on tablets, allowing them to interpret the impact of their decisions and behaviors. In addition, familiarization with the app's features and the digital support provided facilitated their participation and empowerment in self-care.

9	Scopus	10.2196/60758	Caballero Mateos et al. (2025)	Female	Efficacy of a Digital Educational Intervention for Patients With Type 2 Diabetes Mellitus: Multicenter, Randomized, Prospective, 6-Month Follow-Up Study	Spain	Quantitative	I	High quality	The experiences of the study participants were positive, highlighting a preference for applications such as WhatsApp for consulting and sharing information with professionals, regardless of their educational and socioeconomic status. In addition, it was observed that communication via videoconferencing was particularly preferred by patients with higher levels of education.		The article mentions that there are still many barriers to the adoption of digital tools in clinical practice, including issues related to patient acceptance and use of technologies, as well as possible limitations in access or digital skills, although no specific barriers are specified in relation to the study participants.	Facilitators for participants in the use of mobile applications for managing type 2 diabetes included the availability of different communication channels tailored to their needs and preferences, such as WhatsApp, videoconferencing, phone calls, and support from a Digital Diabetes Coach supervised by a multidisciplinary team. In addition, flexibility in platforms and the possibility of interacting in different formats (chat, audio, video) facilitated patient participation and engagement.

10	Pubmed	10.1016/j.gerinurse.2022.04.006	Cheng et al. (2022) WeChat platform	Female	Perceptions of Chinese older adults with type 2 diabetes mellitus about self-management mobile platform: A qualitative study	China	Qualitative	III	High quality	The experiences of participants in the study on the use of mobile applications for managing type 2 diabetes were positive, highlighting a better experience when using self-management platforms. Participants particularly appreciated the intuitive interface design and practical features that made it easier to manage their condition. In addition, the platform tailored to their specific needs helped improve their perception and willingness to use these technologies for self-management.	Participants' perceptions of the use of mobile applications for managing type 2 diabetes were generally positive. Older adults valued a better experience when using self-management platforms, highlighting practical features and trust in the system. They also expressed the importance of support from others and the need for platforms to be tailored to their needs to facilitate their use and increase their willingness to use them regularly.	Participants faced several barriers to using mobile applications to manage their type 2 diabetes, including difficulties related to cognitive and physical abilities, as well as obstacles related to perception and psychological aspects. The complexity of the platforms, information overload, the presence of advertisements and pop-ups, and uncertainty about the risks of the internet hindered their experience and effective use of these technologies.	Participants found facilitators in the use of mobile applications for managing their type 2 diabetes, such as designs tailored to their needs, practical features, and the possibility of communication and reminders on platforms such as WeChat. In addition, the integration of features that allow for the management of health information and the perception that these platforms can improve their self-management contributed to facilitating their use.

11	Scopus	10.2196/jmir.8712	Desveaux et al. (2018)	Female	A Mobile App to Improve Self-Management of Individuals With Type 2 Diabetes: Qualitative Realist Evaluation	Canada	Qualitative	III	Good quality	The experiences of the study participants indicated that the use of the mobile application for managing type 2 diabetes was perceived as useful for improving self-management, increasing awareness of their condition, and facilitating the monitoring of their health goals. However, they also faced challenges related to data entry, lack of sustained interest, and the need for more integrated and user-friendly features. Overall, they valued the usefulness of the app, but noted that its effectiveness depended on personal motivation and social support.	Participants' perceptions of the use of mobile applications for managing type 2 diabetes were generally positive, highlighting that the app helped them monitor their diet, stress, and glucose levels, increasing their awareness and sense of responsibility. Data visualization allowed them to see positive results between medical visits and motivated greater engagement with the app over time, in addition to strengthening the connection with their healthcare providers.	Participants faced barriers related to data entry, lack of sustained interest, and the need for more integrated and user-friendly features in mobile applications for managing their type 2 diabetes.	Participants found mobile applications that included educational resources, personalized messages, and graded levels of content to be helpful, which helped increase their confidence and motivation to manage diabetes. In addition, the app promoted incremental learning and self-efficacy, facilitating the adoption of behavioral changes.

12	Pubmed	10.1186/s13098-024-01414-9	Dsouza et al. (2024)	Female	Identification of challenges and leveraging mHealth technology, with need-based solutions to empower self-management in type 2 diabetes: a qualitative study	India	Qualitative	III	High quality	The experiences of the study participants indicated that some found benefits in using mobile applications to manage their type 2 diabetes, such as increased confidence in controlling glucose levels, adherence to medication, and improvements in dietary decisions. However, they also expressed difficulties, such as confusion about what actions to take when glucose levels rise and a lack of health literacy, which affected their ability to manage the disease effectively.	Participants' perceptions of the use of mobile applications for managing type 2 diabetes were mostly positive, highlighting benefits such as greater confidence in controlling glucose levels, better adherence to medication, and more informed dietary decisions. However, they also expressed concerns, such as confusion about what actions to take when glucose levels rise and a lack of health literacy, which made effective management difficult.	Participants faced several barriers to using mobile applications to manage their type 2 diabetes, including a lack of knowledge about using smartphones and wearables, limited technological skills, and socioeconomic factors that influenced their ability to adopt these technologies. In addition, some had not previously used mobile health applications, which also represented a barrier to their effective implementation.	Participants found mobile applications to be helpful, as they enabled more effective self-monitoring of blood glucose levels, recording of dietary, physical activity, and medication data, and receiving reminders to follow up on their treatment. Furthermore, the perception that these apps can empower individuals to better manage their condition also facilitated their acceptance and use

13	Pubmed	10.2196/40420	Feng et al. (2023)	Female	The Effectiveness of an eHealth Family-Based Intervention Program in Patients With Uncontrolled Type 2 Diabetes Mellitus (T2DM) in the Community Via WeChat: Randomized Controlled Trial	China	Quantitative	I	High quality	The WeChat-based intervention significantly improved glycemic control in patients with type 2 diabetes, reducing HbA1c levels compared to the control group that received usual care. In addition, participants in the intervention group demonstrated improvements in self-care activities and risk perception, suggesting that the intervention was effective in promoting positive changes in disease management.			

14	Pubmed	10.1016/j.jbi.2015.11.008	Georgsson y Staggers (2016)	Male	An evaluation of patients' experienced usability of a diabetes mHealth system using a multi-method approach	United States	Qualitative	II	Good quality		Participants' perceptions of the use of mobile applications for managing type 2 diabetes were mostly positive. Most participants considered the use of information technology and mobile applications in their healthcare to be a positive development and thought it was beneficial for their self-management of diabetes. In addition, the study found that participants were familiar with the regular use of technology, which facilitated their interaction with the applications and contributed to a favorable perception of their usefulness in controlling the disease.		Participants perceived the use of information technology and mobile applications as a positive and beneficial development for their diabetes self-management, which facilitated their interaction with digital tools. In addition, prior familiarity with computers, the Internet, and mobile phones helped them feel more comfortable and confident in using these applications.

15	Scopus	10.22605/RRH4326	Hall et al. (2018)	Female	Service user preferences for diabetes education in remote and rural areas of the Highlands and Islands of Scotland	United kingdom	Mixed	II	Good quality			The main barriers identified by participants in relation to the use of mobile applications and technology for managing type 2 diabetes were the perception that traveling to attend face-to-face sessions was an obstacle, especially for those who had difficulty getting around or worked hours that were incompatible with the sessions. In addition, some participants, particularly those who did not use the internet, showed resistance or satisfaction with their current situation and did not express interest in learning more through technology, which can also be considered a barrier in terms of acceptance and motivation to use mobile applications.	

16	Scopus	10.2196/56276	Hawkes et al. (2024)	Male	Service Users' Experiences of a Nationwide Digital Type 2 Diabetes Self-Management Intervention (Healthy Living): Qualitative Interview Study	United kingdom	Qualitative	III	High quality	The experiences of participants in the article on the use of mobile applications for managing type 2 diabetes were mostly positive, highlighting the usefulness and acceptance of these tools for self-management. Participants valued the ease of access, the possibility of receiving personalized feedback, and the interactivity offered by the applications, which facilitated monitoring of their condition and promoted greater commitment to self-care. However, they also pointed out the importance of apps being integrated with other technologies and offering relevant content tailored to their needs in order to maintain interest and long-term adherence.	Participants' perceptions of the use of mobile applications for managing type 2 diabetes were generally positive, highlighting the usefulness of these tools in facilitating self-management and access to relevant information. Users valued ease of use, the possibility of receiving personalized feedback, and integration with other technologies they use in their daily lives, which promoted greater commitment to self-care. They also noted that apps offering tailored content and interactive features helped maintain their interest and adherence in the long term.	The barriers faced by participants in using mobile applications for managing type 2 diabetes included difficulties related to accessibility and interaction with the technology, as well as the need for applications to be more personalized and user-friendly in order to maintain long-term engagement. In addition, some participants noted that the lack of adequate support in their local environment could limit the effective use of these tools, and that integration with other technological systems was important to facilitate self-management.	Participants identified several facilitators for the use of mobile applications in the management of type 2 diabetes, including ease of access and the convenience of having information and functions available on their phones, which promotes daily self-management. In addition, they valued interaction with existing technologies and the possibility of receiving personalized feedback, which increased their motivation and commitment to using the applications. The integration of interactive features and personalization also facilitated long-term adherence.

17	Pubmed	10.1177/19322968221148756	Hermanns et al (2024)	Male	Evaluation of a Digital Health Tool for Titration of Basal Insulin in People With Type 2 Diabetes: Rationale and Design of a Randomized Controlled Trial	Germany	Prospective, multicenter, randomized controlled parallel trial	I	High quality	The experiences of the study participants indicated that using the mobile app facilitated basal insulin titration, improved treatment adherence, and helped them achieve their target blood glucose and HbA1c levels more easily. In addition, some participants reported an improvement in their overall well-being and confidence in managing diabetes, without experiencing severe hypoglycemic events.	Participants' perceptions of the mobile app were positive, as they reported greater ease in insulin titration, better adherence to treatment, and a feeling of greater control over their disease. In addition, some participants experienced an improvement in their overall well-being and confidence in managing diabetes, with no reports of severe hypoglycemic events.	Participants faced barriers related to limited proficiency in the use of digital technologies, such as a lack of computer skills, especially among older patients or those with cognitive difficulties. These barriers may limit the effectiveness of digital interventions in certain patient groups.	Facilitators for participants in the use of mobile applications included the ability to perform simpler and more accurate insulin titration, improved treatment adherence, and the possibility of more effective real-time blood glucose monitoring. In addition, ease of communication with healthcare professionals and reduced workload in diabetes management also facilitated their use.

18	Pubmed	10.2196/mhealth.6666	Horner et al. (2017)	Female	Designing Patient-Centered Text Messaging Interventions for Increasing Physical Activity Among Participants With Type 2 Diabetes: Qualitative Results From the Text to Move Intervention	United States	Qualitative	III	Low quality	The experiences of the study participants were generally positive regarding the use of mobile applications for managing type 2 diabetes. Participants expressed satisfaction with the program, considering the text messages to be educational, informative, and motivational. In addition, the use of pedometers and daily step information served as motivational reminders and generated a sense of responsibility and connection to their healthcare center. However, there were also frustrations related to the automation of the program, such as repetitiveness, predictability in delivery schedules, and lack of personalization and interactivity in the messages.	Participants' perceptions of the use of mobile applications for managing type 2 diabetes were mostly positive. Participants found the text messages to be educational, informative, and motivational, and felt that the use of pedometers and daily step information helped maintain motivation and create a sense of accountability and connection to their healthcare center. However, they also expressed frustrations related to the automation of the program, such as repetitiveness, predictability in delivery schedules, and lack of personalization and interactivity in the messages.	Participants expressed frustration with the automation of the text messaging program, specifically due to its repetitiveness, predictable sending schedules, and lack of personalization and interactivity in the messages.	Participants found the text messages to be educational, informative, and motivational, which facilitated their participation and positive perception of the program. In addition, the use of pedometers and daily step information served as a motivational reminder and generated a sense of responsibility and connection to their healthcare center, which facilitated the management of their disease.

19	Scopus	10.2196/37737	Hu et al. (2022)	Female	A Social Media–Based Diabetes Intervention for Low-Income Mandarin-Speaking Chinese Immigrants in the United States: Feasibility Study	United States	Quantitative	II	Good quality	The experiences of the participants in this article indicated that the use of a social media platform for type 2 diabetes self-management education was acceptable and promising. Participants reported high video viewing rates and satisfaction with the program, suggesting that the intervention was well received and feasible for improving diabetes-related outcomes and associated health behaviors. However, it was also noted that data interpretation should be done with caution due to the small sample size and potential biases in self-reported responses.)	Participants' perceptions of the use of mobile applications for managing type 2 diabetes were positive, as they found the intervention acceptable and useful for improving their self-management and diabetes-related outcomes. In addition, they reported high satisfaction with the program and an interest in continuing to use similar platforms in the future.	The article mentions that potential barriers for participants include concerns related to digital literacy and familiarity with mobile technologies, although in general most participants showed interest and ability to use platforms such as WeChat. In addition, it notes that the use of free and widely used platforms can minimize concerns about digital literacy and facilitate program scalability.	Facilitators for participants included high ownership of smart devices and frequent use of the WeChat messaging platform, which is widely used in the Chinese immigrant community. This facilitated acceptance and use of the intervention, as well as reducing concerns related to digital literacy and familiarity with technology

20	Pubmed	10.1371/journal.pone.0299799	Hu et al. (2024)	Female	Feasibility of a family-oriented mHealth intervention for Chinese Americans with type 2 diabetes: A pilot randomized control trial	United States	Randomized controlled trial	I	High quality				Facilitators identified in the study include that most Chinese American participants owned smartphones and frequently used text messaging, which facilitated participation in the mHealth-based intervention. In addition, familiarity with messaging apps such as WeChat and willingness to receive support through simple technologies contributed to the acceptance and feasibility of the approach.

21	Scopus	10.1177/22799036231186338	Jafar et al. (2023)	Female	Enhancing knowledge of Diabetes self-management and quality of life in people with Diabetes Mellitus by using Guru Diabetes Apps-based health coaching	Indonesian	Quantitative	I	High quality	The experiences of the study participants were positive in terms of using the mobile app for managing type 2 diabetes. They reported improvements in their knowledge of self-care, their quality of life, and the adoption of healthy habits. Virtual interaction, through messages and calls, allowed them to assess their condition, receive instructions, and set goals, which contributed to effective learning and greater motivation for self-care.	Participants' perceptions of the use of mobile applications for managing type 2 diabetes were positive. They reported that the application helped improve their knowledge of self-care, their quality of life, and the adoption of healthy habits. In addition, they valued virtual interaction, which included messages and calls, as an effective way to assess their condition, receive instructions, and set goals, which facilitated their learning and motivation for self-care.	The main barriers faced by participants were irregular internet connectivity, which caused interruptions or failures in calls and in the use of the application, especially at night, resulting in incomplete or forgotten missions. In addition, challenges related to resistance to change, technological anxiety, and lack of confidence in using devices for self-care were mentioned, particularly in rural or low socioeconomic areas.	The facilitators that participants had for using mobile applications in managing type 2 diabetes included the availability of virtual interaction, such as messages and calls, which allowed them to assess their condition, receive instructions, and set goals, thus facilitating learning and motivation for self-care. In addition, familiarity with technology and interest in managing their health using mobile devices also acted as facilitators, especially in a context where the application provided continuous feedback and support.

22	Scopus	10.1186/s13098-019-0480-4	Jeffrey et al. (2019)	Male	Mobile phone applications and their use in the self-management of Type 2 Diabetes Mellitus: a qualitative study among app users and non-app users	Australia	Qualitative	III	Good quality	The experiences of study participants indicated that most found that mobile applications improved their self-management of type 2 diabetes and their overall health. Recommendations of the apps by healthcare professionals and positive interactions with them increased satisfaction. Some participants felt that the apps helped them identify trends in their blood glucose levels related to food, calculate carbohydrate content, and centralize their measurements, which made it easier for them to manage their condition.	Participants' perceptions of the use of mobile applications for managing type 2 diabetes were mostly positive. Most felt that the apps improved their self-management and health, and they valued features such as visual representation of trends, ease of navigation, and convenience. However, they also pointed out barriers such as lack of awareness of apps as health tools, connectivity limitations in rural areas, perceptions of the severity of their disease, and levels of technological and health literacy	The barriers identified by participants in the use of mobile applications for managing type 2 diabetes included technical problems, such as poor app design, difficulty navigating, and malfunctions, as well as a lack of technological familiarity, especially among older people, which made it difficult for them to use and understand the applications. In addition, in rural populations, data connectivity also represented a significant barrier, limiting the use of certain offline app features.	Facilitators identified by participants included visual representation of trends, intuitive navigation, and convenience, such as the discretion and portability of the apps. In addition, recommendations of the apps by healthcare professionals and positive interactions with them also facilitated their use. Familiarity with technology and positive attitudes toward it also helped users consider and continue using the apps.

23	Scopus	10.2196/37429	Karimi et al. (2023)	Female	Participants' and Health Care Providers' Insights Regarding a Web-Based and Mobile-Delivered Healthy Eating Program for Disadvantaged People With Type 2 Diabetes: Descriptive Qualitative Study	Australia	Qualitative	III	High quality			The article mentions that the barriers faced by participants in using mobile applications for managing type 2 diabetes include difficulties related to accessing and using digital technologies, especially in socioeconomically vulnerable groups. It also notes that participation may be affected by cultural, linguistic, and socioeconomic barriers, as well as a possible lack of digital skills or motivation to use these tools.	The article points out that facilitators for the use of mobile applications in adults with type 2 diabetes include the perception that these tools are accessible, easy to use, and can improve self-management of the disease. In addition, social support and motivation, as well as the integration of applications into daily routines, also facilitate their adoption and effective use.

24	Scopus	10.2196/12965	Koot et al. (2019)	Male	A Mobile Lifestyle Management Program (GlycoLeap) for People With Type 2 Diabetes: Single-Arm Feasibility Study	Singapore	Quantitative	I	High quality	The experiences of the study participants indicated that the use of the GlycoLeap mobile app was perceived as useful and accessible for managing their type 2 diabetes. Participants valued the ease of use, availability of online educational resources, and health coaching support, which facilitated their participation and engagement with the program. However, some expressed that motivation and adherence varied, and that interaction with the app could be improved to maintain long-term interest.	Participants' perceptions of using mobile apps to manage their type 2 diabetes were generally positive. Participants valued the ease of use, accessibility, and usefulness of the app's features, such as online lessons and health coaching support. However, some expressed that motivation and engagement varied, and that interaction with the app could be improved to maintain long-term interest.		Facilitators for participants in the use of mobile applications for managing type 2 diabetes included the ease of use of the application, access to online educational resources, and health coaching support, which facilitated their participation and engagement with the program.

25	Scopus	10.1177/19322968231174037	Krall et al. (2023)	Female	Mobile Applications to Support Diabetes Self-Management Education: Patient Experiences and Provider Perspectives	United States	Quantitative	III	Good quality	The experiences of the study participants were positive regarding the use of the mobile application for managing type 2 diabetes. Most found the app easy to use (81.4%), visually appealing (85.2%), and useful in terms of content (81.5%), especially the articles (92.6%). In addition, 85.1% of participants reported that they would recommend the app to another person with diabetes.	Participants' perceptions of the use of mobile applications for managing type 2 diabetes were mostly positive. Most found the app easy to use (81.4%), visually appealing (85.2%), and useful in terms of content (81.5%), especially the articles (92.6%). In addition, 85.1% of participants reported that they would recommend the app to another person with diabetes.		Facilitators for participants in the use of mobile applications for managing type 2 diabetes included the perceived usefulness of the content, ease of use, and personalization of the app experience. In addition, the availability of features such as data logging, reminders, and the ability to share information with providers facilitated adoption and satisfaction with the tool.

26	Pubmed	10.17925/EE.2024.20.1.10	Krall et al. (2024)	Female	Insights into the Feasibility and Acceptability of a Mobile Insulin Titration Application in Clinical Practice and Its Effects on Diabetes Outcomes	United States	Quantitative	II	Good quality	The experiences of study participants were positive regarding the use of the mobile application for managing type 2 diabetes. Patients and providers reported high levels of satisfaction and favorable experiences, noting that the app was considered a better option for insulin titration compared to other traditional methods, and that it helped providers feel more confident in providing titration instructions. In addition, patients expressed satisfaction with the convenience and effectiveness of the system, and providers said they would use the app again with their patients.	Participants' perceptions of the mobile app for managing type 2 diabetes were very positive. Patients and providers reported high levels of satisfaction, noting that the app was considered a better option for insulin titration compared to other traditional methods, and that it helped providers feel more confident in providing titration instructions. In addition, providers said they would use the app again with their patients, and patients felt confident using the tool.	The article mentions that patients faced barriers related to their psychosocial needs, problem-solving skills, and health literacy level. These barriers may affect their ability to effectively use the mobile app and follow insulin titration recommendations.	Facilitators identified in the study include satisfaction and positive experiences among both patients and providers, who considered the app to be a better option for insulin titration and felt it helped providers feel more confident when giving instructions. In addition, collaboration with healthcare professionals, such as diabetes specialists, and support from the medical team facilitated effective use of the app and promoted confidence in its use.

27	Scopus	10.1177/14604582241291522	Kuo et al. (2024)	Female	Barriers to mobile personal health assistant in patients living with diabetes	Taiwan	Quantitative	II	Good quality	The experiences of the study participants showed that, although they initially had a positive intention to use the mobile app to manage their type 2 diabetes, this attitude declined after one month of use. However, positive correlations were found between intention to use and factors such as satisfaction, performance expectancy, trust, attitude, and facilitating conditions, suggesting that these aspects influence acceptance and experience with the technology.	Participants' perceptions of the use of mobile applications for managing type 2 diabetes showed that, although they initially had a positive attitude and were willing to use them, this willingness decreased after one month of use. In addition, factors such as satisfaction, performance expectations, trust, attitude, and enabling conditions influenced their intention to use these applications, suggesting that initial perceptions may change over time and with experience.	Participants faced several barriers to using mobile applications to manage their type 2 diabetes, including a decline in intention to use after one month, despite an initial positive attitude. In addition, factors such as satisfaction, performance expectations, trust, attitude, and enabling conditions influenced their acceptance and use of the technology, suggesting that these aspects may hinder sustained adoption of the apps.	Participants perceived that enabling conditions, such as technical support and the availability of adequate resources, positively influenced their intention to use mobile applications to manage their type 2 diabetes. These factors facilitated the adoption and continued use of the technology, although their impact varied according to individual experience and perception.

28	Pubmed	10.2196/25958	Lauffenburger et al (2021)	Female	Preferences for mHealth Technology and Text Messaging Communication in Patients With Type 2 Diabetes: Qualitative Interview Study	United States	Qualitative	III	High quality	The experiences of the participants in the article show that, although some expressed some hesitation about using mobile technology for managing type 2 diabetes, in general, they have incorporated or are in the process of incorporating these tools into their routine. Some mentioned that technology can be useful in supporting self-care activities, but they also pointed out barriers such as lack of social support or discomfort in sharing their condition with family members who judge them. In addition, some participants do not feel the need for reminders about healthy habits, such as eating well and exercising, as they already have knowledge about these topics.	Participants' perceptions of the use of mobile applications for managing type 2 diabetes varied. Some expressed that applications can be useful in supporting self-care activities and improving medication adherence, especially if they are simple and easy to integrate into their daily routine. However, they also showed some hesitation, noting that they prefer positive, personalized communication, and that not everyone feels comfortable sharing their condition or fully trusting technology. Overall, they value simplicity and integration of apps, but want them to be tailored to their individual preferences.	Participants in the study identified several barriers to using mobile apps for managing type 2 diabetes. These included financial concerns related to the cost of apps and access to smartphones, as well as the need for greater simplicity and ease of use in order to integrate them into their daily routine. They also expressed some hesitation or uncertainty about trusting the technology and the perception that apps may not be sufficiently personalized or relevant to their specific needs.	Participants identified several facilitators for the use of mobile applications in managing type 2 diabetes. Among these, they highlighted the simplicity and ease of integrating the technologies into their daily routine, which helps prevent burnout and facilitates the monitoring of their self-care activities. They also valued the fact that the applications provide reminders and positive support, which can motivate them and improve adherence to treatments and healthy habits. In addition, familiarity with the use of mobile phones in their daily lives acts as a facilitator for adopting these tools in their disease management.

29	Scopus	10.1186/s12889-021-11552-8	Leon et al (2021)	Female	Process evaluation of a brief messaging intervention to improve diabetes treatment adherence in sub-Saharan Africa	Malawi and South Africa	Randomized controlled trial	III	High quality	Participants' experiences with using text messages to manage type 2 diabetes were mostly positive. Participants found the intervention acceptable and useful, reporting improvements in their health, such as more stable and controlled blood sugar levels. Some mentioned that the messages helped them maintain adherence to their medication, even in difficult situations, such as side effects or fatigue, and that the timely reminders prevented them from forgetting to take their medication. However, participation and level of engagement varied; some felt they did not need the messages, while others recognized clear benefits in their behavior and health.	Participants' perceptions of the use of text messages for managing type 2 diabetes were mostly positive. Participants found the intervention acceptable and useful, reporting improvements in their health, such as more stable and controlled blood sugar levels. Some mentioned that the messages helped them adhere to their medication and remember to take it, even during difficult times. However, participation and engagement varied, and some felt they did not need the messages, although they recognized potential benefits for others	The barriers faced by participants included the perception that text messages alone were not sufficient to improve clinical and behavioral outcomes, suggesting that digital interventions need to be accompanied by other health system supports and broader self-management strategies. In addition, some participants may have experienced difficulties related to motivation, ability, or opportunities to follow recommendations, although this is not specifically detailed in the abstract	The facilitators identified in the study include the perception that the text messages were useful and easy to understand, which motivated participants to follow the recommendations and feel supported in managing their diabetes. In addition, accessibility and familiarity with mobile phones facilitated participation, as did the perception that the intervention could improve their glycemic control and adherence to treatment

30	Pubmed	10.2196/31449	Leong et al (2022)	Female	Social Media–Delivered Patient Education to Enhance Self-management and Attitudes of Patients with Type 2 Diabetes During the COVID-19 Pandemic: Randomized Controlled Trial	Taiwan	Randomized controlled trial	I	High quality	The experiences of study participants indicated that those with a high workload had difficulty improving their self-care activities, such as eating high-fat meals. In addition, the use of videos and messages on the social platform helped to foster positive attitudes and increase knowledge about diabetes, although changing self-care behaviors was more challenging and required a more individualized, patient-centered approach .	Participants' perceptions of using mobile apps to manage their type 2 diabetes were generally positive, as interventions using videos and messages on social media platforms helped foster positive attitudes and increase knowledge about the disease. However, some participants, especially those with heavy workloads, found it difficult to improve their self-care activities, such as eating a high-fat diet, indicating that commitment and motivation are important factors for the successful use of these technologies	One of the barriers identified in the study was that participants with a high workload found it difficult to improve their self-care activities, such as maintaining a healthy diet and reducing their consumption of high-fat foods. This suggests that work stress and lack of time may limit patients' ability to follow recommendations and take advantage of mobile applications for managing their diabetes	Participants found that the use of videos and messages on social media platforms facilitated knowledge acquisition and fostered positive attitudes toward diabetes management, which served as a facilitator for their participation and engagement with the intervention

31	Pubmed	10.1111/jnu.12667	Lewinski et al. (2021)	Female	Perceptions of Using Multiple Mobile Health Devices to Support Self-Management Among Adults With Type 2 Diabetes: A Qualitative Descriptive Study	United States	Qualitative	III	Good quality	The experiences of the study participants indicated that they found the use of mobile devices such as an activity tracker, a wireless glucometer, and a scale with cellular connectivity to monitor their health data for six months to be feasible, useful, and acceptable. In addition, some participants mentioned that these devices provided reminders that served as encouragement and positive reinforcement for their self-care behaviors. The use of mobile technology also facilitated conversations with their healthcare providers, family, and friends. Overall, individual perceptions and skills influenced the usefulness and acceptance of these tools for managing type 2 diabetes	Participants' perceptions of the use of mobile applications for managing type 2 diabetes were mostly positive. Participants considered mobile devices, such as activity trackers, wireless glucometers, and cell-connected scales, to be feasible, useful, and acceptable for monitoring their health data over six months. In addition, some mentioned that the devices provided reminders that served as encouragement and positive reinforcement for their self-care behaviors. They also appreciated that the technology facilitated conversations with their healthcare providers, family, and friends. Overall, individual perceptions and skills influenced the usefulness and acceptance of these tools for managing their disease	Participants faced challenges related to data overload, fatigue, and a lack of understanding of the information collected by mobile devices, which could lead them to abandon their use because they felt overwhelmed or confused	Facilitators identified in the study include the perception that using multiple mobile devices was feasible and useful for supporting type 2 diabetes self-management, as well as the availability of data visualizations that helped understand and monitor the disease. In addition, prior familiarity with the technology and the ability to access and use the collected data also facilitated its use and acceptance

32	Pubmed	10.2196/37534	Li et al. (2022)	Female	Community Health Worker-Led mHealth - Diabetes activado Auto - administración Educación y Apoyo Intervención en Adultos Latinos Rurales: Single-Arm Feasibility Trial.	United States	Quantitative	I	High quality	Participants were very satisfied with the use of mobile applications to treat their type 2 diabetes and used mHealth technologies regularly and frequently throughout the study. Although they had little previous experience with mHealth and low expectations regarding its impact on diabetes control, most began using the applications in the first week and continued to do so consistently throughout the study period. In addition, they reported that the technologies did not pose a significant barrier to their self-care and showed improvements in their expectations of the benefits of mHealth and their health-related digital literacy	Participants were very satisfied with the use of mobile applications to manage their type 2 diabetes and used mHealth technologies regularly and frequently during the study. Although they had little experience with these technologies and low expectations about their effectiveness, most began using the applications during the first week and continued to do so consistently. In addition, they reported that the technologies did not pose a significant barrier to their self-care and showed improvements in their expectations about the benefits of mHealth and their health-related digital competence	Participants reported having little prior experience with mHealth and low levels of health and eHealth literacy, which can be considered a barrier. However, despite these difficulties, most began using the apps in the first week and maintained consistent use throughout the study, indicating that these barriers did not prevent their participation or use of the technologies	Participants found that mHealth technologies facilitated self-management and remote monitoring of their diabetes, as well as promoting interaction with community workers, which contributed to greater satisfaction and engagement with the intervention

33	Scopus	10.3390/electronics13193862	Liu y Yu, 2024	Female	Development of a T2D App for Elderly Users: Participatory Design Study via Heuristic Evaluation and Usability Testing	China	Mixed	II	Good quality	The experiences of the participants in the article indicate that, although mobile applications have the potential to improve the management of type 2 diabetes, older users show less commitment and satisfaction due to usability issues, such as information overload, lack of interactivity, and difficulties in data visualization. In addition, it is highlighted that older users face specific challenges related to their limitations and needs, which affect their participation and adherence to the use of these applications	The perceptions of the participants in the article indicate that, although mobile applications have the potential to improve the management of type 2 diabetes, older adults face difficulties related to usability, such as complex interfaces, unrecognizable icons, and monotonous designs, which reduce their engagement and satisfaction with these tools. In addition, adapting to new technologies and interpreting information are challenges that affect their experience and willingness to use the apps effectively .	The main barriers faced by participants in using mobile applications to manage type 2 diabetes include usability-related difficulties, such as complex interfaces, unrecognizable icons, monotonous designs, and the dispersion of information in dense layouts, which reduces their engagement and satisfaction. In addition, age-related cognitive and physiological limitations, such as vision, hearing, coordination, and memory problems, hinder effective interaction with these applications	The facilitators that participants had for using mobile applications in the management of type 2 diabetes include the design of simple interfaces with easy navigation, recognizable icons, and layouts that reduce cognitive load, as well as interactive features and visual feedback that improve the user experience. These aspects help motivate and facilitate the adoption and continued use of applications by older adults

34	Scopus	10.2196/25151	Maharaj et al. (2021)	Female	Comparing Two Commercially Available Diabetes Apps to Explore Challenges in User Engagement: Randomized Controlled Feasibility Study	Australia and New Zealand	Randomized controlled trial	I	High quality	The experiences of participants in the study on the use of mobile applications for managing type 2 diabetes were mostly positive in terms of initial impressions, the visual appearance of the apps, and the usefulness of features such as glucose level monitoring and medication and food logs. However, they also reported challenges related to usability, such as the apps being slow and complex, and expressed a desire for more educational content, personalized reminders, and features that would make it easier to manage their condition	Participants' perceptions of using mobile apps to manage their type 2 diabetes were mostly positive in terms of the usefulness of features such as glucose level tracking, which they found very useful, and ease of use initially. However, they also expressed that the apps could be slow and complex, and that it would be beneficial for them to include more educational content, personalized reminders, and features that would facilitate the management of their disease	Participants faced several barriers when using mobile applications to manage their type 2 diabetes, including the complexity and slowness of the apps, difficulties in navigation, and problems with features such as calorie and carbohydrate counting. They also reported that the applications could be confusing and that some aspects were difficult to understand or use, which affected their commitment and continuity of use	Participants found mobile applications acceptable initially, as they had favorable impressions and were willing to download them in the future. In addition, they valued useful features such as glucose level tracking and ease of use at first

35	Scopus	10.5603/DK.a2022.0012	Mahoney et al. (2022)	Male	Use of a Diabetes Self-Management Application in Combination with a 4 mm Pen Needle and Its Impact on Glycemic Variability and Patient-Reported Outcomes in People with Type 2 Diabetes Using Basal-Bolus Insulin Therapy	United States	Prospective, multicenter, randomized controlled parallel trial	I	High quality	People with type 2 diabetes who participated in the trial, and who were already using multiple daily doses of insulin, had the experience of incorporating the BD Diabetes Care app into their treatment along with the use of a 4 mm short needle. The experience was marked by a favorable assessment of comfort and less pain during injections, which contributed to a more positive perception of their therapeutic routine. Although the clinical results did not show significant differences, participants reported feeling supported in the process of self-management of their disease.	In terms of perception, most patients expressed satisfaction with the intervention. They highlighted both the usefulness of the mobile app, which offered reminders and educational content, and the practicality of the new needle, which was more comfortable and less painful. Although the level of app usage varied, users recognized that this combination could strengthen their confidence and adherence to insulin treatment.	Among the obstacles identified are low consistency in the use of the application, as interaction time was limited and uneven among participants. The fact that the version of the app used was outdated also had an impact, which may have affected the experience. In addition, the reduction in sample size and the difficulties caused by the COVID-19 pandemic limited the statistical power of the study. Furthermore, the requirement to have modern smartphones restricted the participation of some patients.	Facilitating factors included the ease of use and comfort of the short 4 mm needle, which was considered less painful and better accepted by users, thereby promoting adherence to treatment. Similarly, although used irregularly, the mobile app provided educational and practical support with reminders and records that reinforced self-management. The positive perception of the intervention led to the conclusion that integrating digital resources with more comfortable devices is a promising strategy for optimizing the experience and control of type 2 diabetes.

36	Pubmed	10.2196/37882	Mash et al., 2022	Male	Evaluating the Implementation of the GREAT4Diabetes WhatsApp Chatbot to Educate People With Type 2 Diabetes During the COVID-19 Pandemic: Convergent Mixed Methods Study	South Africa	Mixed	II	Good quality	People with type 2 diabetes who participated in the program were able to interact with a WhatsApp chatbot that sent them educational voice messages related to self-care, healthy eating, physical activity, and COVID-19 prevention measures. Many reported that they were able to improve certain lifestyle habits, mainly in terms of diet, exercise, and adherence to treatment. They also said that the intervention made them feel supported and more motivated to manage their disease during lockdown.	The perception was mostly favorable among both users and healthcare professionals. The vast majority of patients found the messages useful and reported an increase in confidence in managing their condition. For their part, healthcare teams saw the chatbot as a complement to face-to-face education, noting that it saved them time in consultations and made it easier to reach particularly vulnerable patients in the context of the pandemic.	The process faced some challenges, such as technical failures of the tool, which sometimes repeated messages or crashed due to the massive influx of users. Limitations in access to smartphones among certain groups were also identified, as well as a lack of digital skills among older people, which led to dependence on family members or community workers to use the service. Added to this was the initial low motivation of some health professionals to promote the chatbot, due to the high workload resulting from the health crisis.	Among the factors that favored implementation were the population's familiarity with WhatsApp, widespread access to mobile data, and the existence of community support networks. Dissemination through mass media such as radio and television allowed the program's reach to expand rapidly, impacting thousands of users. Likewise, cooperation between the university, the health system, and community organizations strengthened confidence in the intervention and ensured its acceptance as a valid tool for diabetes education and self-care.

37	Scopus	10.1016/j.jcte.2017.07.002	Mathiesen et al., 2017	Female	The influence of diabetes distress on digital interventions for diabetes management in vulnerable people with type 2 diabetes: A qualitative study of patient perspectives	Denmark	Qualitative	III	High quality	Participants described their experience with diabetes as a difficult and exhausting process. Many opted for avoidance strategies, such as justifying their habits or ignoring the disease, which allowed them to cope with daily pressure. However, this attitude generated feelings of guilt and distress that limited their ability to comply with self-care and medical recommendations	Patients stated that digital tools and health messages did not meet their needs or reflect their reality. They found the information they received confusing, contradictory, or irrelevant, which increased their mistrust. In addition, they felt that the use of technology reduced their freedom and did not replace the importance of human contact, so they valued direct guidance from a professional or a close companion more highly	The main obstacles identified were poor understanding of health information, limitations in the use of technological devices, lack of clarity and consistency in educational campaigns, and the absence of strong social support networks. Added to this was the impact of stress, associated illnesses, and the perception that digital applications were invasive and complicated to use in everyday life	The elements that would facilitate acceptance of these interventions were the support of an assigned health professional and the presence of a "support companion" (friend, family member, or colleague) to help control the disease. The possibility of receiving clear, simple, and reliable information was also valued, as was having a more accessible and empathetic care system that understood their limitations and offered constant support

38	Scopus	10.1016/j.invent.2021.100384	Mellergård et al (2021)	Female	Developing a web-based support using self-affirmation to motivate lifestyle changes in type 2 diabetes: A qualitative study assessing patient perspectives on self-management and views on a digital lifestyle intervention	Sweden	Qualitative descriptive	III	High quality	They stated that their work is physically and emotionally demanding due to excessive workloads and a lack of resources. However, they pointed out that they find satisfaction in the care they provide to patients, as they perceive their work to have profound human value and a positive impact on the care process.	Staff feel that, despite their efforts, their role in the healthcare system does not always receive the recognition it deserves. They perceive a disparity between their daily work and the institutional and social appreciation they receive. They also claim that working conditions affect both their well-being and the quality of care they provide.	Among the main obstacles are the lack of appropriate supplies and equipment, job overload, unfair pay, and the lack of space to take care of themselves. Added to this is limited participation in decision-making and poor institutional response, which increases feelings of exhaustion and frustration.	As factors that favor their performance, they mentioned ethical and vocational commitment, cooperation among colleagues, and the support generated through teamwork. Likewise, the gratitude of patients and their families is an important stimulus, as it reinforces their sense of purpose and motivates them to maintain close and humane care despite existing limitations.

39	Scopus	10.1016/j.pec.2021.06.033	Mitchell et al. (2020)	Female	Patient engagement and presence in a virtual world world diabetes self-management education intervention for minority women	United States	Exploratory qualitative	III	Good quality	The doctors stated that their daily work is marked by a heavy patient load and a lack of sufficient resources to provide efficient care. Even with these limitations, they emphasized that serving the community gives them a sense of purpose, which reinforces their vocation and commitment to the profession.	Participants pointed out that the healthcare system does not always provide them with the right conditions to meet the demands of their work. They believe there is a mismatch between the responsibilities they are required to fulfill and the tools available to them. In addition, the lack of recognition, both institutional and social, affects their motivation and their willingness to remain at this level of care	The most frequently mentioned obstacles were excessive workloads, lack of infrastructure and supplies, insufficient numbers of professionals, low pay, and lack of opportunities for academic growth. Added to this is the absence of emotional support and the complexity of administrative procedures, which further hinders the practice of medicine.	Factors that help sustain their performance include a dedication to service, the establishment of close ties with patients, and cooperation with other colleagues. Similarly, the gratitude expressed by the community and recognition within teams were perceived as incentives that strengthen their motivation despite the limitations of the system.

40	Scopus	10.2196/60246	Mueller et al. (2024)	Male	Disease Awareness in Patients With Type 2 Diabetes: Analysis of Baseline Data From the SMART-Finder Observational Study	Germany	Retrospective and prospective observational cohort study	III	Good quality	The experiences of participants in the SMART-Finder study reveal a low level of knowledge regarding biomarkers linked to renal function, such as UACR and eGFR, despite their proficiency with HbA1c. A large proportion of patients were unaware of their eGFR (91.1%) and UACR (94.1%) results, reflecting a perception of poor information and limited communication on the part of healthcare personnel.		The obstacles identified include poor adherence to diagnostic guidelines and limitations in communication between healthcare professionals and patients, coupled with a possible excessive focus on HbA1c control in patient education processes.	Among the possible facilitators are regular diagnostic testing and strengthening communication, with the aim of improving awareness and knowledge about kidney health.

41	Scopus	10.2196/31832	Mueller et al. (2022)	Male	Evaluation of Self-care Activities and Quality of Life in Patients With Type 2 Diabetes Treated With Metformin Using the 2D Matrix Code of Outer Drug Packages as Patient Identifier: the DePRO Proof-of-Concept Observational Study	Germany	Observational, multicenter, cross-sectional	III	Good quality	People with type 2 diabetes who were being treated with metformin participated entirely digitally using the my ePRO app. To authenticate their participation, they scanned the 2D code on their medication and then completed electronic questionnaires related to self-care activities, satisfaction with therapy, and quality of life. The experience was positive, as most participants were able to complete the process without external support and with a very high questionnaire completion rate.	In terms of patient perception, it was observed that they spent on average about half of the week on self-care practices. Most said they were satisfied or very satisfied with their treatment, and a considerable proportion reported good overall health. However, several participants reported episodes of hypo- or hyperglycemia, and more than half reported discomfort related to pain, reflecting a favorable assessment of the treatment but with certain limitations in their daily experience.	Among the main obstacles were low participation rates, especially for remote invitations sent via mobile apps, compared to personal invitations in pharmacies. Other factors included lack of interest, time constraints, lack of smartphones, and restrictions imposed by the COVID-19 pandemic. In addition, mistrust of data security and unfamiliarity with the use of digital tools represented additional barriers.	Factors that favored implementation included the direct invitation from pharmacists, which encouraged greater acceptance; the simple and intuitive design of the application, which made it easy to complete the questionnaires; and the fact that the entire procedure was conducted anonymously and without the need to visit a health center. Added to this were the symbolic financial incentive and the autonomy provided by the digital tool, which facilitated patient participation.

42	Scopus	10.2196/jmir.6601	Muller et al. (2017)	Female	Effects on engagement and health literacy outcomes of web-based materials promoting physical activity in people with diabetes: An international randomized trial	United Kingdom, Austria, Germany, Ireland and Taiwan	Exploratory qualitative	I	High quality	Participants described how COPD affects their daily lives, especially due to limitations on physical activity and the necessary adjustments to their routines. They also described the emotional burden of the disease, as it interferes with their personal autonomy and their ability to maintain full social relationships.	Regarding their perception of the disease, several patients expressed feelings of distress, sadness, and frustration at the progressive evolution of their condition. However, they emphasized that the support of their families and professional care were elements that gave them confidence and hope to better cope with the situation.	The lack of clear information about the disease, poor adherence to prescribed treatments, difficulties in accessing specialized services, and the stigma associated with the constant use of oxygen were identified as obstacles. Added to this are negative perceptions about their quality of life and low motivation to maintain healthy practices on a sustained basis	Among the factors that facilitated coping were close family support, a relationship of trust with healthcare professionals, the availability of respiratory rehabilitation programs, and effective communication with the medical team. These elements helped patients develop greater resilience and better comply with care recommendations.

43	Scopus	10.2196/70203	Newhouse et al. (2025)	Female	Experiences of Using a Digital Text Messaging Intervention to Support Oral Medication Adherence for People Living With Type 2 Diabetes: Qualitative Process Evaluation	United Kingdom	Qualitative	III	High quality	Participants identified comprehensive benefits in using the system, as it generated positive changes in their behaviors, ways of thinking, and attitudes toward diabetes and self-care. Several noted that the SMS messages served as constant reminders, in a subtle and non-invasive way, which promoted adherence to treatment and conveyed the feeling that someone was looking out for their well-being. Some also mentioned that the messages became a source of support and companionship, especially in cases where the relationship with health services was insufficient or unsatisfactory.	The assessment of the messages varied and changed depending on the personal situation and needs of each participant; while some considered that certain content was not immediately useful to them, they still appreciated receiving it for its potential future relevance, and in general the messages helped to reinforce awareness of self-care and encourage greater individual responsibility in managing health.	Adherence to drug treatment was disrupted by changes in daily routine, emotional factors, work and family responsibilities, as well as life situations such as moving house, loss, or health and social problems. In addition, several participants had limited knowledge of their condition and medications, which complicated self-management. The relationship with healthcare professionals, perceived in many cases as distant and paternalistic, generated feelings of judgment, lack of support, and reduced motivation to control diabetes. On the other hand, messages that required interaction were confusing and frustrating, especially when they received automatic errors in response. Likewise, some considered certain messages to be too general or unclear, which made them difficult to apply in practice, while the repetition and monotony of others diminished their impact, especially on those who had already established self-care routines.	Pre-established routines for taking medication promoted adherence, especially when supplemented with reminders or the use of tools such as alarms and pill boxes. The diversity of message content and the repetition of key ideas with different formulations helped to maintain participants' attention and interest. Likewise, the ability to customize the frequency and timing of messages according to individual preferences facilitated their incorporation into daily life. Access to links to reliable sources of information, such as Diabetes UK, met educational needs and stimulated active knowledge seeking. Social support was also highlighted as an essential resource for self-management, whether through the collaboration of family members in feeding or medication reminders. Finally, the consistency of the system over time contributed to the development and maintenance of habits that strengthened adherence and self-care.

44	Scopus	10.2196/diabetes.9059	Öberg et al. (2018)	Female	Perceptions of persons with type 2 diabetes treated in swedish primary health care: Qualitative study on using ehealth services for self-management support	Sweden	Qualitative	III	Good quality	The interviewees recounted their experiences with the use of digital health services such as mobile applications, electronic portals, and virtual forums. They mentioned that these tools helped them become more independent, strengthen their sense of responsibility, increase their commitment to self-care, and feel more confident. In addition, they appreciated that these technologies facilitated communication with healthcare professionals and other people living with diabetes. (Oberg, 2018)	The participants' views were mixed. On the one hand, they highlighted the benefits in terms of empowerment, knowledge acquisition, greater participation, and immediate access to support for managing the disease. But at the same time, they expressed doubts about the reliability of the information found on the internet, concerns about privacy, and a certain insecurity about not being fully proficient with technology (Oberg, 2018).	The main obstacles identified were a lack of digital skills, limited technical support, physical limitations that made it difficult to use smartphones (such as vision problems or tremors), unstable internet connections, and mistrust of online information. There was also concern that digital care would replace direct contact with healthcare professionals, which many perceived as necessary. (Oberg, 2018)	The factors that favored the use of these tools were immediate and continuous access to information and support, the sense of security derived from password-protected and encrypted systems, the possibility of empowerment through greater knowledge and participation in decisions, contact and exchange with other patients through virtual communities, and reduced travel to health centers, which provided them with comfort and freedom (Oberg, 2018).

45	Scopus	10.2196/40377	Orozco-Beltrán et al. (2022)	Male	Effects of a Digital Patient Empowerment and Communication Tool on Metabolic Control in People With Type 2 Diabetes: The DeMpower Multicenter Ambispective Study	Spain	Quantitative	I	High quality	Participants assessed their satisfaction with the DeMpower app and their experience with the healthcare they received using specific questionnaires. Satisfaction with the app was measured using the Diabetes Treatment Satisfaction Questionnaire status (DTSQs), and the healthcare experience was assessed using the Chronic Patient Experience Assessment Instrument (IEXPAC).	User perceptions of their healthcare experience and satisfaction with the DeMpower app were assessed using specific questionnaires, such as the IEXPAC and DTSQs. Participants shared their opinions on the app's usefulness and ease of use, as well as its impact on managing their diabetes and their relationship with healthcare professionals.	The main barriers identified in the study were related to the COVID-19 pandemic, which caused the premature interruption of the study, making it difficult to enroll and follow up with patients, as well as limiting their attendance at medical visits and procedures, which affected the continuity of follow-up and the evaluation of the results.	The facilitators identified in the study include the perception that the DeMpower app can facilitate better disease control and improve communication between patients and healthcare professionals, which can improve diabetes management. Furthermore, most patients positively rated the app's usefulness and ease of use, contributing to its acceptance and continued use.

46	Scopus	10.1093/tbm/ibae034	Piette et al. (2024)	Male	Behavioral Engagement and Activation Model Study (BEAMS): A latent class analysis of adopters and non-adopters of digital health technologies among people with Type 2 diabetes	United States	Qualitative	II	Good quality	The women shared their experiences of attending maternal care at health centers. Although they recognized the importance of receiving institutional care during pregnancy and childbirth, many faced difficulties related to the quality of treatment received, infrastructure deficiencies, and limited availability of resources. However, when the support was respectful and the care timely, their experience became more satisfactory.	The participants' perceptions of maternal services showed varying nuances. On the one hand, they valued the fact that institutional care offered safety and could prevent serious complications. But at the same time, they expressed dissatisfaction with the lack of empathy shown by certain professionals, the long waits, and the shortages in the supply of basic supplies.	The main limitations were related to the long distances to health centers, the costs of transportation and care, the shortage of personnel and material resources, and the unfavorable attitudes of health personnel, which discouraged women from seeking care.	Factors that encouraged the use of services included the existence of community support programs, the support of family members and close networks, and positive interactions with staff who showed empathy and respect. These aspects increased confidence in health services and encouraged the decision to use them during pregnancy.

47	Scopus	10.2196/15744	Poduval et al. (2020)	Male	Feasibility, acceptability, and impact of a web-based structured education program for type 2 diabetes: Real-world study	United Kingdom	Mixed	III	Good quality	The results showed that, although it was possible to offer the program in a real clinical setting, the proportion of people who completed the program was low, with only 9% of those registered completing it. However, those who completed the program significantly improved their perception of control over the disease and reduced their associated emotional distress.	From the participants' perspective, it was evident that working at their own pace, having access to content in various formats—such as texts, images, and videos showing testimonials from people with diabetes—and being able to access education without having to travel were aspects that were positively valued and facilitated the experience. In addition, the emotional support component was highlighted, as many users identified with the emotions expressed in the videos, which alleviated their feelings of isolation.	However, several barriers were identified that led to low adherence to the program. Among them, lack of time due to work and family responsibilities led many users to interrupt their participation. Some patients said they did not feel prepared to receive information at the beginning of their diagnosis or felt that the content did not fit their particular situation, especially those who were not undergoing drug treatment and felt that the material was more relevant to those who were. Others felt that they already had enough knowledge to manage their condition, which also reduced their motivation to complete the course	In terms of facilitating factors, flexible access to the web-based program allowed participants to progress according to their availability, and the varied formats favored different learning styles, reinforcing understanding and interest. In addition, receiving electronic reminders served to motivate continued use of the program. Health professionals, for their part, recognized that one of the limitations to promoting participation was the limited time available during consultations to adequately explain the benefits of structured education. Therefore, they suggested delegating some responsibilities for promoting and registering patients for the program to administrative assistants or nurses, as well as the need to further personalize the content and facilitate access from mobile devices to increase convenience

48	Scopus	10.2196/15789	Prinjha et al. (2020)	Female	British South Asian Patients' Perspectives on the Relevance and Acceptability of Mobile Health Text Messaging to Support Medication Adherence for Type 2 Diabetes: Qualitative Study	United Kingdom	Qualitative	III	High quality	Participants recounted their experiences with type 2 diabetes: difficulties with diet, medication adherence, physical activity, and stress management. They mentioned the role of family in reminding them to take their medication, preparing healthy meals, and accompanying them to medical appointments. They shared experiences with the use or non-use of digital devices and how this influences their self-care	They saw the potential of SMS messages to support self-care, not only in terms of medication but also diet, exercise, and emotional management. Some felt that receiving messages in English would be useful for improving their language skills, while others preferred translated options. They perceived family involvement as necessary for the intervention to be practical.	Technological barrier: older adults without cell phones or with low digital literacy. Language barrier: difficulty reading English or written South Asian languages. Cultural barrier: lack of dietary plans adapted to South Asian food; mistrust of interventions that do not take cultural or religious practices (such as fasting) into account. Acceptance barrier: some expressed that too many messages could be annoying and lead to disinterest.	Family support (children, spouses, caregivers translating or reminding them to take their medication). Culturally adapted SMS messages (content on South Asian foods, fasting, natural remedies, exercise in women's groups). Different message formats (text, audio, images, WhatsApp). In-person groups for those who do not use digital devices. Credibility of the source (messages sent from primary care physicians or the health system).

49	Pubmed	10.2196/10271	Rossmann et al. (2019)	Female	Appropriation of Mobile Health for Diabetes Self-Management: Lessons From Two Qualitative Studies	Singapore and Germany	Qualitative	III	High quality	Patients had varying experiences with diabetes-specific apps, ranging from no use to long-term use. Most patients with type 1 diabetes used diabetes apps, while few patients with type 2 diabetes used them. The use of apps for monitoring (e.g., glucose logs) and nutritional information was reported. Many patients preferred automated systems to facilitate monitoring. (Rossmann, 2019)	In addition to diabetes-specific apps, they used general health apps, messaging apps (such as WhatsApp), and web search engines to obtain information and communicate. In terms of functional perception, participants valued monitoring, nutritional information, and communication with peers. Symbolic perception (social status and sense of agency) appeared in the German study but not in the Singapore study. The relationship with physicians had a normative influence: patients who were dependent on their physicians were reluctant to use apps when their physicians did not recommend them (Rossmann, 2019).	The barriers reported include financial restrictions (not wanting to pay for apps), time restrictions (time required to use apps), cognitive restrictions (lack of knowledge about available apps), and technical restrictions (technical failures, device incompatibilities, small screen size for patients with vision problems, dependence on phone battery). Some patients expressed concerns about data privacy in cloud services. Technology and apps did not always meet their needs, leading to discontinuation of use (Rossmann, 2019).	For some motivated and knowledgeable patients, mobile apps and devices were useful tools that facilitated diabetes monitoring and management. Communication and information exchange with other patients through support groups and messaging apps facilitated use and acceptance. The sense of agency and social status associated with technology use were facilitators in the study in Germany (Rossmann, 2019).

50	Scopus	10.1093/tbm/ibad020	Spierling-Bagic et al. (2023)	Female	Process evaluation of Dulce Digital-Me: an adaptive mobile health (mHealth) intervention for underserved Hispanics with diabetes	United States	Randomized controlled trial	II	Good quality	Participants experienced the process of receiving educational messages, using mobile devices (glucometers, electronic pill dispensers), and having contact with a health coach (HC). In interviews, they said they learned new things about managing diabetes, improved their habits, and valued the support, noting that they would do it again because it provided them with motivation and help.	Most reported positive perceptions: 93% felt that the intervention helped them manage their diabetes better, and 99% would recommend it to family or friends. They rated the messages as “useful,” “motivating,” and “educational.” Some mentioned confusion with the messages in the automated group, which also shows critical perceptions	Difficulties were identified, such as some patients' resistance to changing habits, the presence of stress and depression as frequent obstacles, problems with time to attend or respond, and a certain degree of technological fatigue in the automated group. There were also limitations in the participation of men and older people due to cultural and access factors	Personal motivation, the support of educational messages, and above all, the presence of a bilingual Hispanic health coach with personal experience with diabetes, who inspired trust and closeness. The use of personalized reports that allowed recommendations to be adjusted and questions to be answered quickly. Weekly contact was seen as a key element for adherence

51	Pubmed	10.1016/j.jbi.2023.104481	Sze et al. (2023)	Female	StepAdd: A personalized mHealth intervention based on social cognitive theory to increase physical activity among type 2 diabetes patients	Japan	Study of a pre-post intervention design without a control group.	II	Good quality	The mHealth intervention based on social cognitive theory enabled patients with type 2 diabetes to acquire skills to improve their self-care, especially in managing physical activity, through the use of the StepAdd app to monitor and adjust their daily step goals, resulting in a significant increase in physical activity and improvements in parameters such as HbA1c and BMI.	Patients perceived the intervention as effective and motivating, as personalization and automatic monitoring through the app increased their motivation and commitment to behavioral change. High participation and retention (97%) reflect a positive perception of the use of technology and the support received	Although not explicitly detailed in the abstract, common barriers in this type of intervention include loss of motivation over time, difficulties in adhering to goals, and possible technological or digital literacy limitations. However, in this study, the high retention rate suggests that these barriers were minimal or well managed	Key facilitators were the personalization of the intervention, the use of mobile technology, the support of community pharmacists, and the ability to automatically synchronize data, which facilitated active participation, effective monitoring, and the perception of ongoing support, contributing to the success of the intervention.

52	Pubmed	10.3389/fendo.2025.1420578	Tang et al. (2025)	Male	Factorial design study of self-management using Dnurse App in T2DM patients	China	Randomized controlled trial	I	High quality	Participants mentioned that using the mobile app helped them manage their type 2 diabetes, as it provided reminders, educational resources, and the ability to monitor their condition, which helped them better organize their self-care and gave them a positive perception of the digital support they received. (Tang, 2025)	Participants expressed a favorable assessment of the application, describing it as practical, motivating, and a complementary support to medical care. They also noted that it gave them confidence and encouraged adherence to treatment by making them feel that they were not alone in managing their disease. (Tang, 2025)	Some participants had limited digital skills, which made it difficult for them to use the app smoothly. This was compounded by technical problems such as connection failures or limited internet access, as well as some initial resistance to change on the part of older adults who were unfamiliar with technology. (Tang, 2025)	Facilitating factors included the availability of cell phones as an accessible tool for patients, the personalization of messages and reminders, the support of the healthcare team in integrating the application into clinical management, and the ease of use of the platform, which allowed even older adults to adapt comfortably. (Tang, 2025)

53	Scopus	10.1186/s12913-019-4486-2	Torbjørnsen et al (2019)	Female	Users' acceptability of a mobile application for persons with type 2 diabetes: a qualitative study	Norway	Descriptive qualitative	III	High quality	Positive experiences were reported in relation to ease of use, up-to-date information, simplicity, and the use of language appropriate for users. However, negative experiences were also identified, associated with the complexity of certain applications, difficulty in locating functions, information overload, frustration due to usability issues, and cultural or contextual discrepancies between the app design and the user.	In general, users perceive mobile applications for managing their disease positively, highlighting the usefulness of educational messages, glucose level tracking, monitoring physical activity and diet, as well as support in decision-making for managing their condition. Likewise, healthcare professionals show interest in monitoring and suggesting the use of these tools.	Barriers such as technical difficulties, unfamiliarity with technology, complicated usability, small print or awkward buttons, and semantic or cultural differences in apps developed in other contexts have been identified. These barriers can cause frustration among users.	Factors that facilitate use include the simplicity and intuitiveness of the application, access to information and recommendations in an appropriate language, and the option to share data with healthcare professionals for more effective monitoring. In addition, the motivation and willingness of users to employ these tools despite initial difficulties is highlighted.

54	Pubmed	10.2196/mhealth.8824	Torbjørnsen et al. (2018)	Female	Acceptability of an mHealth App Intervention for Persons With Type 2 Diabetes and its Associations With Initial Self-Management: Randomized Controlled Trial	Norway	Randomized controlled trial	I	High quality	Participants had a positive experience using the diabetes diary app, highlighting benefits such as greater control over their health, greater involvement in their treatment, and complementary support to conventional medical care. In addition, many acquired new skills and techniques for managing the disease, which was associated with a perception of greater benefit, and in some cases, the support of specialist nurses enhanced both the learning experience and the motivation to use the tool.	Most participants considered the app useful and beneficial for improving self-control and self-management of diabetes, with those who had greater self-management skills tending to perceive it more positively. However, when adjusted for age, gender, and frequency of use, the perception of benefit was mainly related to frequent use of the app, and some users also noted that it strengthened communication and the support they received in their medical care.	Technical limitations were identified, as some users encountered difficulties with the technology, were less motivated initially, or had limited ability to learn how to use the application. In addition, digital and health literacy influenced participation, as those with less technological knowledge or understanding of health issues encountered greater obstacles to involvement. Although all participants had experience with mobile phones, not all were familiar with smartphones, which in some cases restricted adoption of the tool.	Initial training and ongoing technical support enabled even participants with limited digital skills to use the app, while telephone support from specialist nurses encouraged motivation to use it and strengthened self-management. Furthermore, frequency of use stood out as the most decisive factor, as those who used the app regularly perceived greater benefits and acceptability. The intrinsic motivation of patients to voluntarily participate in the study was also key, which favored adherence.

55	Scopus	10.1186/s12875-024-02373-w	Tyagi et al. (2024)	Female	Primary Technology Enhanced Care Home HbA1c Testing (PTEC HAT) programme: a feasibility pilot study in Singapore	Singapore	Pilot study of feasibility and acceptance, pre- and post-intervention design, without control group	II	Good quality	Participants reported high satisfaction with the components of the home HbA1c testing program, completing the test with varying levels of assistance from the care team. (Tyagi, 2024)	Most participants perceived benefits in using the home testing kit, considering it useful and convenient for managing their diabetes. (Tyagi, 2024)	The main barrier identified was the difficulty in remembering and completing multiple steps correctly, which increased the cognitive load and hindered the effective use of the kit. (Tyagi, 2024)	The provision of educational resources, such as instructional videos and easy-to-understand guides, was seen as a useful strategy for reducing cognitive load and improving adherence and adoption of the method. (Tyagi, 2024)
56	Scopus	10.2196/67293	Whittemore et al. (2025)	Male	Multilevel Intervention to Increase Patient Portal Use in Adults With Type 2 Diabetes Who Access Health Care at Community Health Centers: Single Arm, Pre-Post Pilot Study	United States	Clinical trial	II	Good quality	Participants received a tablet with internet access for 6 months and were trained to use the patient portal and tablet. The intervention included assessment of social needs and connection to community resources, as well as ongoing support from community health workers (CHWs) and clinical nurses. (Whittemore, 2025)	Support for autonomy as perceived by patients was measured, reflecting how providers promoted self-efficacy and self-care. Patients valued the training and support received, although there was anxiety about privacy and doubts about the usefulness of the portal. (Whittemore, 2025)	The main barriers included low health and technology literacy, lack of regular access to internet-enabled devices, language issues, doubts about the usefulness of the portal, lost passwords, anxiety about viewing medical information, and privacy concerns. In addition, limited integration of the program into clinical care and lack of activated features on the portal made it difficult to use. (Whittemore, 2025)	The support of CHWs in overcoming technological and social barriers, specific training tailored to people with low literacy levels, the use of methods such as "teach-back," and connection to community resources were key facilitators. Support from clinical nurses and the provision of devices with internet access also helped. (Whittemore, 2025)

57	Pubmed	10.1186/s40814-020-0558-7	Whittemore et al. (2020)	Male	Development of a diabetes self-management + mHealth program: tailoring the intervention for a pilot study in a low-income setting in Mexico	Mexico	Qualitative	II	Low quality	While the use of mobile health and text messaging was well received by adults with type 2 diabetes and healthcare professionals, concerns were raised regarding access to cell phones, the ability to access text messages or images, understanding of messages, and continued interest in messages over time. (Whittemore, 2020)	Recommendations included assessing the ability of adults with type 2 diabetes to use text messaging on their personal cell phones, the importance of simplified colloquial language in text messages, and the need to avoid language that could be interpreted as overly prescriptive (e.g., “you need to... you should”). (Whittemore, 2020)	Barriers to proper diabetes management include misguided cultural beliefs about its causes and treatments, such as attributing the disease to fear or stress rather than factors related to diet and physical activity; difficulties in modifying lifestyle, especially in diet and exercise; lack of access to healthy foods, medications, and supplies for disease control; low health literacy and limited socioeconomic conditions that hinder understanding and application of self-care strategies; lack of family support or demands that compete with recommendations; mental health issues such as stress, anxiety, or depression that interfere with self-management; and restrictions on the use of mobile technologies, including difficulties receiving or viewing multimedia messages, downloading content, or understanding certain text messages.	Multimedia messaging (MMS) became standard on cell phones in 2011, allowing users to receive images even if their device did not have a camera. During the focus groups, moderators sent both text messages and images to all participants to assess their reception capabilities. Low- and high-resolution images were sent to analyze whether quality affected the reception of MMS messages. (Whittemore, 2020)

58	Scopus	10.1001/jamanetworkopen.2024.24781	Wieland et al. (2024)	Male	Digital Storytelling Intervention for Hemoglobin A1cControl among Hispanic Adults with Type 2 Diabetes: A Randomized Clinical Trial	United States	Randomized controlled trial	I	High quality	The research was conducted in clinics with high proportions of Hispanic populations in Arizona and Minnesota, involving Hispanic adults with type 2 diabetes and poor glycemic control, through a multicenter, randomized clinical trial. (Wieland, 2024)		Some potential barriers include difficulty maintaining patient participation and engagement, as well as possible limitations in access to technology or specific cultural resources that may affect acceptance of the intervention (Wieland, 2024).	The participation of the Hispanic community, the use of relevant cultural narratives, and the integration of the intervention in clinical settings with a high proportion of Hispanic patients facilitated the acceptance and impact of the study (Wieland, 2024).
59	Scopus	10.1186/s12877-024-05224-6	Ye et al. (2020)	Female	Role perceptions and experiences of adult children in remote glucose management for older parents with type 2 diabetes mellitus: a qualitative study	China	Qualitative	III	High quality	Adult children participate in the remote glucose management of their elderly parents with T2DM, acting as health decision-makers, remote supervisors, health educators, and emotional supports.	The involvement of children in glucose management is seen as beneficial and feasible, improving adherence and confidence in glucose control in older people.	The main barriers include concerns about privacy and security, difficulties in adapting to and learning how to use mobile applications, lack of knowledge about glucose management, and the cost of test strips.	The facilitators are the ease of use of the applications, convenience, digital empowerment, family support, and the perception that remote management is beneficial and viable.

60	Scopus	10.2196/16266	Yang et al. (2020)	Female	Effect of a mobile phone-based glucose-monitoring and feedback system for type 2 diabetes management in multiple primary care clinic settings: Cluster randomized controlled trial	South Korea	Randomized controlled trial, multicenter	I	High quality	Participants showed greater interest in the information technology (IT)-based intervention and were mostly younger than those in the control group. Most clinics were able to use the system independently without significant difficulties, and there were no dropouts during the study, indicating a positive experience with the mobile system for diabetes monitoring. (Yang, 2020)	Survey results indicated favorable changes in satisfaction and motivation related to long-term medication adherence in the intervention group. Previous studies also reported greater effectiveness of mobile management systems in terms of patient satisfaction and adherence. (Yang, 2020)	The barriers identified include low participation among older patients, possibly due to lower digital literacy or socioeconomic status, and variability in the intervention by the medical team, which could lead to bias. In addition, the short duration of follow-up and the focus on urban clinics limit the generalizability of the results. (Yang, 2020)	Facilitators include the system's ease of use, prior training, and support from specialized centers, which enabled clinics to use the system autonomously. The multicenter structure and controlled design helped minimize bias and facilitate the implementation of the system in primary care settings. (Yang, 2020)

61	Scopus	10.2196/12936	Yingling et al. (2019)	Female	An Evaluation of Digital Health Tools for Diabetes Self-Management in Hispanic Adults: Exploratory Study	United States	Mixed	III	Low quality	Participants had little prior experience using T2DM monitoring tools, but nearly all were able to use RT-CGM and the activity tracker for self-management, and more than half recorded food intake on a tablet. Most found these tools to be effective at capturing and communicating data, although initial experience was limited. (Yingling, 2019)	Participants appreciated that monitoring devices support behavioral change and raise awareness to improve diabetes self-management. However, they expressed that the technology must be personalized and tailored to their individual needs to be more effective. (Yingling, 2019)	The main barriers identified were fear, trust, calibration requirements, comfort, and cost. In particular, fear of RT-CGM sensor insertion was highlighted, as well as lack of training and difficulties in using the iPad applications. (Yingling, 2019)	Facilitators include adequate training, customization of tools, social support, data integration, and the presence of a community support center that can resolve technical issues. In addition, the availability of tools in Spanish and the training of community "super users" would facilitate their acceptance and use. (Yingling, 2019)

62	Scopus	10.2196/48310	Yoon (Sungwon) et al. (2024)	Male	Acceptability of Mobile App-Based Motivational Interviewing and Preferences for App Features to Support Self-Management in Patients With Type 2 Diabetes: Qualitative Study	Singapore	Qualitative	III	Good quality	Patients reported that motivational interviewing (MI), both in person and via app, provided them with a space for reflection and a useful reminder to maintain healthy habits. They acknowledged that traditional healthcare systems were fragmented and had little time to attend to them, so MI represented additional support. With the AI-based MI app, several noted that it was easier to share their concerns without feeling judged, which made the experience more comfortable and relaxed. They also appreciated the goal-setting, reminder, progress visualization, and reward features. (Yoon, 2024)	Positive: EM was perceived as a motivating complement to routine medical care, capable of promoting behavioral changes and offering continuous support. The modality used was seen as convenient, accessible, and less intimidating than face-to-face care (Yoon, 2024).<br><br>Ambivalent: Some expressed reluctance for fear of losing autonomy, as they felt capable of self-management. Critical: The lack of human contact was questioned, as it could affect the therapeutic relationship and level of trust (Yoon, 2024).	Desire for autonomy in self-management (resistance to external interventions).<br><br>Lack of time and competing demands (family, work).<br><br>Limited digital literacy, especially among older adults, which made it difficult to adopt the application.<br><br>Absence of human contact and nonverbal emotional cues that strengthen the patient-professional relationship.<br><br>Cognitive load when interacting with complex applications or those with excessive features. (Yoon, 2024)	Convenience and flexibility of use (can be used anywhere, anytime).<br><br>Personalized content (advice tailored to diet, medication, or physical activity).<br><br>Anonymity and less perceived judgment, allowing for more open communication.<br><br>Motivational features: goal setting, progress tracking, interactive reminders, gamification, and rewards.<br><br>Hybrid model: combining app automation with support from human health coaches to build trust and resolve complex questions. (Yoon, 2024)

63	Scopus	10.3390/ijerph192215415	Yoon (Sungwon) et al. (2022)	Male	Personal Goals, Barriers to Self-Management and Desired mHealth Application Features to Improve Self-Care in Multi-Ethnic Asian Patients with Type 2 Diabetes: A Qualitative Study	Singapore	Qualitative	III	High quality	Participants shared that the use of mHealth applications can be useful, but they face difficulties related to the frequency of notifications and the routine of data entry, which can be an impediment to their effective use. (Yoon, 2022)	Patients believe that app features such as personalized reminders and social support can improve their self-management if tailored to their individual and cultural needs. The general perception is that these technologies have the potential to facilitate diabetes management. (Yoon, 2022)	The main barriers identified include difficulties in adhering to regular meal times, limited healthy food options at work, physical problems that hinder physical activity, and work schedules that make it difficult to adhere to medication and follow regimens (Yoon, 2022).	

64	Scopus	10.1177/14604582251317914	Shen et al. (2025)	Female	Diabetes apps cannot "stand alone": A qualitative study of facilitators and barriers to the continued use of diabetes apps among type 2 diabetes	China	Qualitative	III	High quality	Users' experiences with diabetes management and app usage vary depending on their level of knowledge and familiarity. Novice users find that the app provides inconsistent information and is useful at first, but as they gain more knowledge and experience, they become experts in managing their condition and consider the app no longer necessary, as they feel they have complete control and the information becomes repetitive.	Users perceive the app as a useful tool for monitoring their health and feeling secure, especially when tracking their glucose levels and recording their daily diet. However, some consider the information in the app to be repetitive and lacking in novelty, which reduces its perceived value and usefulness over time.	App-specific barriers: Inconsistent information and lack of updates, which causes confusion and leads users to abandon the app, especially when they feel they have mastered the management of their diabetes.<br>Barriers related to user experience: Increased knowledge and experience leads users to consider it unnecessary to continue using the app, as they feel they have reached a level of mastery in their self-care.<br>Social and cultural barriers: Social perception and stigma, such as difficulty using the app at festivals or in social situations where users feel that using the app may cause others to judge them or make them feel different, as well as difficulty talking openly about diabetes for fear of negative comments	Concern for health and glucose control, which motivates users to record data and search for information in the app to maintain their well-being [5][9].<br>Personal responsibility and the desire to manage their condition effectively, even in social situations where alcohol or food consumption can be a challenge.

65	Pubmed	10.3390/ijerph19031902	Zamanillo-Campos et al. (2022)	Female	Patients' Views on the Design of DiabeText, a New mHealth Intervention to Improve Adherence to Oral Antidiabetes Medication in Spain: A Qualitative Study	Spain	Qualitative	III	High quality	Patients shared experiences about how they manage their diabetes: difficulties in following their diet, experiences taking medication while traveling or at social gatherings, use of pill boxes, mobile apps, smart scales, among others. They also recounted their daily experience with the disease, describing it as “silent” and difficult to perceive.	They expressed opinions about medication (some trusted it, others were wary of taking many pills), about diet (recognized as important, but with practical and social limitations), and about physical activity (perceived as necessary, but difficult to maintain). They also expressed their positive perception of the DiabeText intervention, considering it useful and acceptable in general.	Various barriers to diabetes management were identified, including a lack of clear and sufficient information about the disease and self-care, overuse of medication and forgetting to take drugs, difficulties in accessing medication due to the deadlines set by pharmacies, economic and social limitations that make it difficult to maintain a proper diet, lack of motivation, time, or confidence to engage in physical activity, and problems using mobile phones, which constitutes a technological barrier.	Various support elements were identified that facilitate diabetes management, including the use of reminders such as pill boxes, alarms, and text messages; social support provided by family and friends; the availability of clear, personalized information requested by patients; motivation generated through positive and practical messages; and the use of technological tools such as mobile applications, smart scales, and educational SMS messages.

66	Scopus	10.3389/fpubh.2023.1259158	Zhang et al. (2023)	Female	Factors affecting the willingness of patients with type 2 diabetes to use digital disease management applications: a cross-sectional study	China	Cross-sectional type	I	High quality	Experience with diabetes management applications (DMAs) can influence acceptance and use. Familiarity with technology and understanding of its benefits facilitate adoption, especially in older populations who may have difficulty with the interface and technical use	Key perceptions include perceptions of safety and usefulness. Patients consider the safety and reliability of applications to be fundamental to their use, as well as the perception that these tools improve control of their disease. The perception of usefulness has a direct impact on willingness to use these applications, being more highly valued than ease of use.	The main barriers identified are concerns about the security of personal information, the risk of data loss, and system vulnerabilities. In addition, usability-related difficulties, such as difficulty learning how to use the application or understanding its functions, also represent obstacles, especially for older users with potential physical or cognitive limitations.	Perceived security (PS) includes concerns about personal data protection and confidence in the reliability of the system. Perceived ease of use (PEOU) reflects how much users believe they can learn and use the application without difficulty. Perceived usefulness, although not directly mentioned in the specific sections, is inferred from the importance patients attach to specific benefits, such as effective glucose control, and the influence this has on their willingness to use the applications.
