# Supplementary material for: User experiences and perceptions on the use of digital health technologies in the management of type 2 diabetes: an integrative systematic review
Source: Front Clin Diabetes Healthc. 2026 Feb 3;7:1750055. doi: 10.3389/fcdhc.2026.1750055 (PMC12909238; doi:10.3389/fcdhc.2026.1750055)
Supplement: Supplementary file 3 [file DataSheet3.pdf]

Supplementary File 1: Complete Search Strategies and Results

Electronic Database Searches

Search Dates

Searches were conducted on the following dates:

- **PubMed (via MEDLINE):** 23 August 2025
- **Scopus:** 28 August 2025
- **Google Scholar:** 31 August 2025

1. PubMed

Complete Search String:

("Diabetes Mellitus, Type 2"[MeSH] OR "type 2 diabetes") AND ("Mobile Applications"[MeSH] OR "mobile app*" OR "mHealth" OR "smartphone application") AND ("User-Centered Design"[MeSH] OR "Perception" OR "Attitude" OR "barriers" OR "facilitators" OR "acceptability" OR "satisfaction") AND ("Self-Management" OR "disease management" OR "chronic disease management") Filters: English, Spanish, Humans, last 10 years

MeSH Terms Used:

- Diabetes Mellitus, Type 2 [MeSH]
- Mobile Applications [MeSH]
- User-Centered Design [MeSH]

Filters Applied:

- **Languages:** English, Spanish
- **Species:** Humans
- **Publication dates:** Last 10 years (2015-2025)
- **Article types:** All types included

Search Results:

- Total records retrieved: n = 104

Interface Used:

PubMed Advanced Search Builder (https://pubmed.ncbi.nlm.nih.gov/)

2. Scopus

Complete Search String:

(TITLE-ABS-KEY (type 2 diabetes) AND TITLE-ABS-KEY (mobile app) OR TITLE-ABS-KEY (mhealth app) OR TITLE-ABS-KEY (digital health) AND TITLE-ABS-KEY (user experience) OR TITLE-ABS-KEY (perceptions) OR TITLE-ABS-KEY (attitude to health) OR TITLE-ABS-KEY (facilitators AND barriers) OR TITLE-ABS-KEY (acceptability) OR TITLE-ABS-KEY (patient satisfaction) AND TITLE-ABS-KEY (self management) OR TITLE-ABS-KEY (disease management)) AND PUBYEAR > 2014 AND PUBYEAR < 2026 AND (LIMIT-TO (OA , "all")) AND (EXCLUDE (LANGUAGE , "German") OR EXCLUDE (LANGUAGE , "French") OR EXCLUDE (LANGUAGE , "Portuguese"))

Search Fields:

- TITLE-ABS-KEY: Searches in article title, abstract, and author keywords

Filters Applied:

- **Publication years:** 2015-2025 (PUBYEAR > 2014 AND PUBYEAR < 2026)
- **Access type:** Open Access (all types)
- **Languages excluded:** German, French, Portuguese
- **Languages included:** English, Spanish (and other languages not explicitly excluded)

Search Results:

- Total records retrieved: **n = 202**

Interface Used:

Scopus Advanced Search (https://www.scopus.com/)

3. Google Scholar

Complete Search String:

("type 2 diabetes" OR "diabetes tipo 2") AND ("mobile app*" OR "aplicaciones móviles" OR mHealth OR "salud digital")

Bilingual Strategy:

The search included terms in both English and Spanish to capture:

- Literature from Latin American contexts
- Spanish-language publications from Spain and Latin America
- Regional publications potentially not indexed in PubMed or Scopus

Search Procedure:

- **Results ordered by:** Relevance (Google Scholar default ranking)
- **Pages screened:** 1 page (first results page)
- **Total results reviewed:** 12 articles
- **Date range:** 2015-2025 (set in Google Scholar interface)

Rationale for Limited Scope:

Google Scholar was used as a supplementary source to PubMed and Scopus for several reasons:

1. The highly specific combination of search terms (diabetes + digital health + user experience) naturally limits relevant results
2. PubMed and Scopus provide comprehensive coverage of peer-reviewed literature in health sciences
3. Google Scholar's primary value in this review was to capture potential grey literature, regional publications, or preprints not indexed in the main databases
4. The first page of Google Scholar results (ranked by relevance) captures the most pertinent publications matching the search criteria

Search Results:

- Total records screened: **n = 12**

Interface Used:

Google Scholar (https://scholar.google.com/)

Supplementary Search Methods

1. **Citation Tracking**

Method:

- Forward citation tracking: Identified articles citing each included study
- Backward citation tracking: Reviewed reference lists of included studies
- Tools used: Google Scholar "Cited by" feature and Scopus citation tracking

Results: Forward and backward citation tracking was systematically performed for all 66 included studies. This process did not identify additional articles that met the inclusion criteria within the specified publication period (2015-2025). Several potentially relevant articles were identified but were excluded because they:

- Were published outside the date range (pre-2015)
- Focused on type 1 diabetes or gestational diabetes
- Did not specifically address user experiences/perceptions
- Were conference abstracts or protocols without full empirical data

2. **Reference List Screening**

Method: Reference lists of all 66 included studies were manually screened to identify additional relevant publications that may have been missed in the database searches.

Results: No additional articles meeting the inclusion criteria were identified through reference list screening. Most references in included studies were either:

- Already captured in our database searches
- Published before 2015
- Focused on clinical outcomes rather than user experiences
- Review articles or theoretical papers

3. **Hand Searching**

Method: Systematic hand searching of specific journals was not performed.

Rationale: Comprehensive database searches (PubMed and Scopus provide broad coverage of health sciences literature) combined with citation tracking and reference screening were deemed

sufficient to capture the relevant literature. The inclusion of Google Scholar also helped identify any significant publications outside traditional indexing systems.

Search Results Summary Table

Database	Initial Retrieval	After Title/Abstract Screening	After Full-Text Review	After Deduplication	Final Included*
PubMed	104	47	27	9	21
Scopus	202	62	78	17	45
Google Scholar	12	12	0	0	0
Citation tracking	0	0	0	0	0
Reference screening	0	0	0	0	0
**TOTAL**	318	121	105	26	66

**Note: The final column shows the number of studies from each source that were included in the final review. Some studies appeared in multiple databases and are counted in each source where they were retrieved.*

Notes on Search Strategy Development

Controlled Vocabulary (MeSH Terms):

MeSH (Medical Subject Headings) terms were used in PubMed to ensure comprehensive retrieval:

- **"Diabetes Mellitus, Type 2"** captures all articles indexed with this condition
- **"Mobile Applications"** includes articles about apps, mobile software, and smartphone applications
- **"User-Centered Design"** encompasses user experience, usability, and human-centered approaches

Free-Text Terms and Wildcards:

- **Wildcards (*):** Used to capture word variations (e.g., "app*" retrieves app, apps, application, applications)
- **Quotation marks (""):** Used for exact phrase searching (e.g., "type 2 diabetes")
- **Truncation:** The asterisk allows for retrieval of all word endings

Boolean Operators:

- **AND:** Used to combine different concepts (diabetes AND technology AND user experience AND self-management)
- **OR:** Used within concepts to capture synonyms and related terms (e.g., "mobile app" OR mHealth OR "smartphone application")

Language Considerations:

The inclusion of Spanish terms in Google Scholar was strategic:

1. Aligns with the review's objective to include diverse geographical contexts
2. Captures literature from Latin America and Spain
3. Addresses the noted gap in representation from low- and middle-income countries
4. Reflects the reality that important regional research may be published in Spanish

Search Term Selection Process:

Search terms were selected based on:

1. Preliminary scoping searches to identify relevant vocabulary
2. Analysis of keywords in highly relevant articles
3. Consultation with medical librarians (if applicable)
4. Pilot testing of search strings to optimize sensitivity and specificity

Search Sensitivity vs. Precision:

The search strategy was designed to favor sensitivity (comprehensive retrieval) over precision (high proportion of relevant results), which is appropriate for systematic reviews. This approach ensures that relevant studies are not inadvertently missed, though it requires more extensive screening of results.

Quality Assurance

Peer Review of Search Strategy:

The search strategies were reviewed by JFP, RCSB, and MTMC, nurses and members of the research team, to ensure their completeness and accuracy prior to implementation.

Search Documentation:

All searches were documented with:

- Exact date of search

- Complete search strings as entered
- Number of results retrieved
- Any filters or limits applied
- Interface/platform used (including version where applicable)

Reproducibility:

This supplementary file provides sufficient detail to allow independent researchers to replicate the searches and verify the results, consistent with PRISMA 2020 reporting standards.

References for Search Methodology

- Page MJ, McKenzie JE, Bossuyt PM, et al. The PRISMA 2020 statement: an updated guideline for reporting systematic reviews. *BMJ* 2021; 372: n71.
- Rethlefsen ML, Kirtley S, Waffenschmidt S, et al. PRISMA-S: an extension to the PRISMA Statement for Reporting Literature Searches in Systematic Reviews. *Syst Rev* 2021; 10: 39.

Contact Information

For questions regarding the search strategy or to request additional information, please contact:
Judith Francisco-Pérez judithfrancisco@gmail.com
